# The double-edged sword of inflammation in inherited retinal degenerations: Clinical and preclinical evidence for mechanistically and prognostically impactful but treatable complications

**DOI:** 10.3389/fcell.2023.1177711

**Published:** 2023-04-13

**Authors:** Kubra Sarici, Aanal Vyas, Alessandro Iannaccone

**Affiliations:** Duke Center for Retinal Degenerations and Ophthalmic Genetic Diseases, Duke Eye Center, Department of Ophthalmology, Duke University School of Medicine, Durham, NC, United States

**Keywords:** inherited retinal degeneration (IRD), inflammation, immune activation, treatment, prognosis

## Abstract

We present retrospective data from our clinical research efforts of the past several years alongside a review of past and current clinical and preclinical data independently by several investigators supporting our clinical evidence for the importance of inflammation in inherited retinal degenerations (IRDs). We show how inflammation is a complicating factor in IRDs but, if recognized and managed, also a great opportunity to mitigate disease severity immediately, improve patient prognosis and quality of life, extend the treatment windows for gene-specific and agnostic therapeutic approaches, mitigate the impact of inflammatory complications on the accurate estimate of vision changes in IRD natural history studies, improve the chances of safer outcomes following cataract surgery, and potentially reduce the likelihood of inflammatory adverse events and augment the efficacy of viral vector-based treatment approaches to IRDs. Manuscript contribution to the field. Inflammation has been suspected to be at play in IRDs since the beginning of the 1900s and became a research focus through the early 1990s but was then largely abandoned in favor of genetic-focused research. Thanks to regained cognizance, better research tools, and a more holistic approach to IRDs, the recent reappraisal of the role of inflammation in IRDs has brought back to the surface its importance. A potential confounder in natural history studies and a limiting factor in clinical trials if not accounted for, inflammation can be managed and often offers an opportunity for immediately improved prognosis and outcomes for IRD patients. We present our retrospective clinical evidence for connections with a measurable secondary autoimmune component that can develop in IRDs and contribute to vision loss but is at least in part treatable. We also present ample lines of evidence from the literature corroborating our clinical observations at the preclinical level.

## Introduction

Inherited retinal degenerations (IRDs) comprise a genetically and clinically heterogeneous group of conditions due to mutations in over 300 distinct genes characterized by the common leitmotif causing the progressive degeneration of photoreceptors and vision loss (Daiger, 2020). The mechanisms of degeneration in IRDs have been under investigation for decades and remain under further characterization. The presence of clinically visible inflammatory changes in IRD patients has been noted since the early days of the field, leading to the development of the term “*retinitis* pigmentosa” to characterize RP, the most common of the IRDs. The most apparent clinical change related to ongoing inflammation at the tissue level is the presence of cystoid macular edema (CME), a well-known and common complication of IRDs. In addition to CME, virtually every IRD specialist has seen IRD patients also present with retinal exudates, perivascular sheathings, vascular staining, and/or leakage of the far peripheral vessels or at the vascular arcades on intravenous fluorescein angiography (IVFA). It has been our experience that far less appreciated appear to be signs of late leakage on IVFA at or around the optic nerve head and swelling of the retinal nerve fiber layer (RNFL) on optical coherence tomography (OCT)—but it has been our experience that they are also present. The less frequent appreciation of these features is in part since inflammatory disc changes in IRDs tend to be best appreciated in the very late IVFA frames (>6 min), and that glaucoma specialists or neuro-ophthalmologists routinely ask for RNFL OCTs, but not by IRD—or more in general, retinal—specialists.

The presence of these inflammatory findings in IRDs led researchers in the field to investigate, in the late 1980s, the immune system reactivity against retinal antigens in patients with RP (Chant et al., 1985; Detrick et al., 1985; Heckenlively et al., 1985; Chan et al., 1986; Detrick et al., 1986; Percopo et al., 1990). In early studies, in addition to immune cellular activation, high levels of anti-retinal auto-antibodies (AR-AAbs) were also found in various cohorts of RP patients (Brinkman et al., 1980; Heckenlively et al., 1985; Broekhuyse et al., 1988). Furthermore, CME was shown independently by several investigators to be associated with elevated AR-AAb levels (Heckenlively et al., 1996; Heckenlively et al., 1999; Wolfensberger et al., 2000). In RP, it also shown that CME can be treated with carbonic anhydrase inhibitors (CAIs) administered either orally, topically, or both (Marmor, 1990; Grover et al., 1997; Wolfensberger, 1999; Grover et al., 2006; Apushkin et al., 2007), the “fluid-draining” ability of which is due to the expression of membrane-bound carbonic anhydrase II (CA-II) in the RPE (Wolfensberger et al., 1994). It suggested that responsiveness to CAIs may be linked to the presence of anti-CA-II AAbs, which are common in RP patients with CME (Wolfensberger et al., 2000). However, there can be rebound effects from prolonged treatment or following discontinuation (Apushkin et al., 2007), since CAIs do not address any underlying inflammatory component, as noted in uveitis patients with CME (Schilling et al., 2005). Therefore, in the uveitis subspecialty world, in which the inflammatory etiology of CME is well established and accepted, CME is far more commonly treated with topical steroids, non-steroidal anti-inflammatory drugs (NSAIDs), in conjunction with oral steroids, various types of steroid-sparing immuno-modulating treatment (IMT) regimens such as mycophenolate mofetil (MMF), methotrexate (MTX), azathioprine (AZT), and more recently a variety of biologic agents such as adalimumab, with or without subtenon, intravitreal and—more recently also—suprachoroidal steroid injections or injectable/implantable steroid slow-releasing devices (Steinmetz et al., 1991; Tanner et al., 1998; Tranos et al., 2004; Androudi et al., 2005; Jain et al., 2005; Perry and Donnenfeld, 2006; Hariprasad and Callanan, 2008; Hogewind et al., 2008; Jones and Francis, 2009; Slabaugh et al., 2012; Wu et al., 2012; Bourgault et al., 2013; Koop et al., 2013; Rossetto et al., 2015; Sen et al., 2015; Grixti et al., 2016; Asproudis et al., 2017; Feiler et al., 2017; Frere et al., 2017; Juthani et al., 2017; Khurana et al., 2017; Pichi et al., 2017; Doycheva et al., 2018; Petrushkin et al., 2018; Schallhorn et al., 2018; Hasanreisoglu et al., 2019; Ansari et al., 2021; Saade et al., 2021; Wong et al., 2021; Chronopoulos et al., 2022; Studsgaard et al., 2022; Miguel-Escuder et al., 2023). These remedies have been used successfully to manage CME in RP patients who are refractory or incompletely responsive to CAIs (Forte et al., 1994; Heckenlively et al., 1999; Saraiva et al., 2003; Kim, 2006; Scorolli et al., 2007; Park et al., 2013; Ahn et al., 2014; Patil and Lotery, 2014; Lemos Reis et al., 2015; Schaal et al., 2016; Sudhalkar et al., 2017; Bakthavatchalam et al., 2018; Mansour et al., 2018; Karasu, 2020; Park et al., 2020; Veritti et al., 2020; Chen et al., 2022), and this has also been our experience thus far.

The early investigations into the role of the immune system in RP pathogenesis and progression led us to investigate the therapeutic potential of an IMT agent available in the 1990s, thymopentin (Rispoli et al., 1990; Rispoli et al., 1991; Vingolo et al., 1993a; Vingolo et al., 1993b; Iannaccone et al., 1994a). Administered i.v., thymopentin reduces immune system activation. In brief, in an open-label, prospective, pilot trial comparing automated visual field (VF) sensitivity and retinal function by virtue of the mixed full-field flash electroretinogram (ffERG) b-wave amplitudes to a historical natural history control group (Berson et al., 1985), serial intravenous thymopentin administration improved both parameters at 18 and 36 mos (Rispoli et al., 1990; Rispoli et al., 1991; Vingolo et al., 1993a; Vingolo et al., 1993b; Iannaccone et al., 1994a) vs. both declining progressively in the historical control group (Berson et al., 1985). These results supported the hypothesis that immune system activation is a contributing factor to IRD pathobiology and progression. Unfortunately, this drug is no longer available on the market, yet numerous IMT regimens are possible nowadays.

Over 3 years have gone by since the time when the role of the immune system in IRDs was being first investigated, and this hypothesis was set aside as the focus of IRD researchers rapidly shifted towards the discovery of new genes and the characterization of genotype-phenotype correlations. More recently, though, there has been a progressive reappraisal of the role of inflammation and activation of the immune system in IRDs (Iannaccone et al., 1994b; Iannaccone et al., 1995; Epstein et al., 2014; Epstein et al., 2015; Hollingsworth et al., 2015; Iannaccone et al., 2015; Hollingsworth et al., 2017; Iannaccone et al., 2017; Adamus, 2018; Hollingsworth et al., 2018; McMurtrey and Tso, 2018; Iannaccone and Radic, 2019; Birch et al., 2020; Duncan et al., 2020; Hollingsworth and Gross, 2020; Liu et al., 2020; Yang et al., 2020; Adamus, 2021; Alekseev et al., 2021; Iannaccone et al., 2021; Alekseev et al., 2022; Funatsu et al., 2022; Gupta et al., 2022). Several lines of evidence will be reviewed further in the discussion section to provide additional support to immune-mediated inflammation representing a potential treatment target for IRDs (Adamus et al., 2004; Xiong et al., 2013; McMurtrey and Tso, 2018; Hollingsworth and Gross, 2020; Funatsu et al., 2022), including work from our group (Epstein et al., 2014; Hollingsworth et al., 2015; Gattegna et al., 2019; Iannaccone and Radic, 2019). Independently, also two IMTs commonly used to manage inflammatory eye disease, including autoimmune retinopathy and/or optic neuropathy (AIR/AINR), MMF and MTX, have been shown to have potential treatment effects on animal models of RP (Liu et al., 2020; Yang et al., 2020), whereby further investigations on these drugs are in progress and intravitreal MTX is already being tested in a human clinical trial of RP (NCT05392179, Aldeyra Therapeutics). While these specific drugs have exhibited mechanistic effects on specific aspects of the retinal degenerative process that make them attractive as treatments for RP, MMF also clearly showed a normalization of the intraretinal microglial activation patterns (Yang et al., 2020). Therefore, the effects of MMF and MTX on RP-induced inflammation cannot be ignored and may be in part responsible for some of the observed benefits.

Some investigators have questioned the potential pathogenicity of AR-AAbs, and the need for standardized testing methods has been emphasized (Fox et al., 2016). In prior studies, we have found that control subjects, too, can exhibit autoreactivities. (Iannaccone et al., 2012; Iannaccone et al., 2015; Iannaccone et al., 2017). Thus, the presence of AR- or ON-AAbs is not automatically diagnostic of AIR/AINR. Autoreactivities need to be interpreted in the context of the entire clinical picture before considering initating treatment. A consensus on the diagnostic approaches to AIR/AINR has been reached and more refined testing standards have been instituted (Fox et al., 2016; Adamus, 2020). Notwithstanding these caveats, the pathogenicity of AR-AAbs implicated in AIR/AINR and its paraneoplastic form, cancer-associated retinopathy (CAR), has been extensively characterized and confirmed (Adamus et al., 1997; Adamus et al., 1998a; Adamus et al., 1998b; Kyger et al., 2013; Xiong et al., 2013; Adamus, 2018). We further showed previously that as many as 60%–70% of AIR and CAR patients exhibit also an anti-optic nerve AAb (AON-AAb) associated optic neuropathy phenotype (i.e., AINR or CARON) independent of being paraneoplastic or not (Adamus et al., 2011). Treating these conditions with steroids and various IMT regimens has been shown to lead to disease mitigation, halting, or even partial reversal (Adamus et al., 2012; Davoudi et al., 2017; Heckenlively and Lundy, 2018a; Finn et al., 2020; Grewal et al., 2021).

Despite all these lines of evidence, detection of the very same AR- or AON-AAbs is not presently considered equally relevant in IRDs, and is most often dismissed as a mere after-the-fact secondary marker of prior degeneration. However, our clinical experience with many IRD patients has been quite different. IRD patients exhibiting inflammatory complications virtually invariably present also with AR- and/or AON-AAb patterns that correlate quite well with clinical and functional findings, especially at the retinal immunohistochemistry (rIHC) testing level, and respond favorably to the treatment of these complications. This is in line with a pathogenic view of these AAbs also in IRDs (Adamus, 2021). Thus, we sought out to conduct a systematic retrospective review of our patients who presented with signs and symptoms of a possible IRD and also exhibited inflammatory signs and, thus, underwent AAb and rIHC testing between 2016 and 2022. We will present data on the incidence of AR- and/or AON-AAbs in IRDs, and show that some genotypes appear to be more commonly associated with these secondary autoimmune reactions. We will illustrate in-depth analyses of these associations in certain genetic subgroups that are a) quite common and b) of enhanced interest because of ongoing natural history studies (RUSH2A, NCT03146078, and Pro-EYS, NCT04127006), and we will illustrate some representative examples of IRD patients with very clear improvements in visual function following treatment of these immune-mediated complications. Far from claiming that IRDs are outright autoimmune disorders, our data will show that the proposed pathogenicity and prognostic relevance of these AAbs is well supported, falls in line with preexisting evidence going back to the 1980s, advocates for increased efforts to tackle these treatable aspects in IRDs, and paves the way for additional avenues to ensure that there is indeed a light at the end of the tunnel for IRD patients.

## Methods

We conducted a retrospective analysis, approved by the Duke Institutional Review Board, of subjects seen at the Duke Center for Retinal Degenerations and Ophthalmic Genetic Diseases who had been referred for a possible diagnosis of IRD but also exhibited inflammatory signs and symptoms, thus, underwent AAb and rIHC testing between 2016 and the summer of 2022 according to published methods (Adamus et al., 2004; Adamus, 2020). The study adhered to the ethical principles of the Helsinki Declaration. Given the retrospective nature of the study, written informed consent was not required.

Patients meeting clinical or imaging criteria that we defined as suggestive or outright indicative of inflammatory complications during their examination (Table 1) underwent a complete eye examination inclusive of best corrected visual acuity (BCVA) to ETDRS charts, VF and ffERGs according to ISCEV (McCulloch et al., 2015) standards (Espion3 system, Diagnosys LLC, Lowell, MA, United States). VFs were most often of the semiautoamated kinetic (SKP) type (Octopus 900 Pro; Haag-Streit AG; Koeniz, Switzerland). When appropriate to better characterize the patterns of VF loss, supplemental VFs with photopic or scotopic (Dark-Adapted Cromatic Perimetry, Medmont Int PTY LTD.; Nunawading, Australia) full-field testing static approaches were also obtained. ERG testing included also, when appropriate, supplemental recordings to assess the specific integrity of the ON- and OFF pathways and the photopic negative response (PhNR), an ffERG photopic response originating from the RGCs (Viswanathan et al., 1999; Frishman et al., 2018). Imaging studies included on every subject macular linear and volume spectral domain optical coherence tomography (SD-OCT, Spectralis, Heidelberg Engineering, Heidelberg, Germany) fundus autofluorescence (FAF), and color fundus photography. FAF and photos were obtained in the vast majority of cases with Optos Wide-Field imaging devices (California model or prior versions). Based on medical indication and necessity when optic nerve involvement was apparent or suspected, a more selected subset of patients underwent also peripapillary (PP) ON SD-OCTs to estimate the thickness of the PP RNFL, assessment of optic nerve functional status via pattern-reversal visual evoked potentials (PVEPs) and IVFAs, typically extended in duration (at least 6–8 min from the injection of the fluorescent dye). In these patients, retinal SD-OCT segmentation analysis of the RGC layer was also usually conducted to ascertain the selective integrity of the RGCs and correlate it as appropriate to PVEP, PhNR, rIHC and ON-AAb test results.

**TABLE 1:** Visual function and clinical/imaging criteria used to identify subjects with IRD-like presentations associated with inflammatory complications.

*Clinical/imaging criteria associated with suspected inflammatory complications*	*Visual function criteria associated with suspected inflammatory complications*
• Absence of waxy pallor on DFE	• Late-onset visual function loss or sudden acceleration in the vision loss process
• Presence of outright overt disc hyperemia and/or swelling on DFE	• BCVA less than potential predicted by foveal EZ preservation not explained by media opacities or other factors (e.g., amblyopia)
• Thickened RNFL on OCT imaging (macular and/or PP scans)	• Worse VF loss than predicted by the level of ffERG reduction
• Late leakage and/or staining on FA of the disc, arcades, macula, peripheral vascular, focal or disseminated	• Asymmetry in VF loss not explained by other factors
• Presence of significant CME (especially if unresponsive/only partially responsive to CAIs)	• Enlarged blind spots or centro-cecal scotomas on VF testing not associated with PP atrophy or other PP chorioretinal lesions
• Disseminated and/or flame-shaped retinal exudates on DFE (as frequently seen in primary and paraneoplastic AIR and AIR/ARRON patients)	• Delayed pattern VEPs despite normal or reasonable (20/25–20/30) BCVA
• Disseminated chorioretinal peripheral nummular “punched-out” atrophic lesions on DFE	• Electronegative ffERGs with evidence of post-receptoral dysfunction and/or (when measurable) disproportionate RGC-driven response (PhNR) reduction

DFE, dilated fundus examination; RNFL, retinal nerve fiber layer; OCT, optical coherence tomography; PP, peripapillary; FA, fluorescein angiography; CAIs, carbonic anhydrase inhibitors; AIR, autoimmune retinopathy; ARRON, autoimmune-related retinopathy and optic neuropathy; BCVA, best corrected visual acuity; EZ, ellipsoid zone; VF, visual field; VEPs, visual evoked potentials; ffERGs full-field electroretinogram; RGC, retinal ganglion cells.

All subjects meeting the above clinical and functional criteria also underwent CLIA-certified diagnostic testing for AAb/rIHC at the Ocular Immunology Laboratory at the Casey Eye Institute, Oregon Health and Sciences University, Portland, OR, meeting updated and refined testing standards as of 2018 (Fox et al., 2016; Adamus, 2020). Tests obtained prior to these new standards by Western blot (WB)-based methods showed specific reactivities only when possible (Adamus et al., 2004). AON-AAb testing was and remains exclusively WB-based, as it is directed against a lysate of a retrobulbar portion of the optic nerve (Adamus et al., 2011). We also strived to obtain in all subjects rIHC, which is performed on normal human donor eye thin retinal sections that are then stained with the patients’ sera and then counterstained with a secondary fluorescent anti-IgG antibody to detect and quantitate reactivities (Adamus et al., 2004). We found rIHC to be especially important to identify the subcellular localization of autoreactivities at the tissue level and help us establish better correlates with clinical and functional findings. While depending on the type and location of the autoreactive epitopes, a patient may be positive for AAb testing but not for rIHC and *vice versa*. In the vast majority of cases, AAb-positive patients were also positive for rIHC testing which allowed us to establish such cellular level correlates with precision. For example, a patient presenting with inflammatory optic nerve findings (disc swelling on SD-OCT and leakage on IVFA) may well present without AON-AAbs but exhibit AR-AAbs and RNFL and/or RGC staining on rIHC, whereas one with mild if any disc changes but exhibiting PVEP delays suggestive of an inflammatory optic nerve component may present exclusively with AON-AAbs and no AR-AAbs and no RNFL and/or RGC staining on rIHC. Also, patients presenting with more complex pictures such as electronegative ffERG due to selective or prevailing b-wave compromise would most often exhibit retinal bipolar cell (BC)/inner nuclear layer (INL) and/or outer plexiform layer (OPL) staining and/or AR-AAbs directed against known BC/INL- and/or OPL-expressed antigens—or even against arrestin, a protein of the visual cycle that, when compromised genetically like in Oguchi disease (Fuchs et al., 1995; Nakazawa et al., 1997), is associated with electronegative ffERG responses. A similar effect on the ffERG response has also been observed when the partner visual cycle protein, rhodopsin kinase, is compromised in Oguchi disease (Yamamoto et al., 1997; Cideciyan et al., 1998; Khani et al., 1998; Yoshii et al., 1998).

All patients who presented not only with inflammatory findings but also a positive family history of an IRD, a clinical picture strongly suggestive of an IRD, or a longtime history of visual symptoms (e.g., night blindness since birth or early childhood) suggestive of a likely IRD, and/or a diagnosis of an RP or macular dystrophy—e.g., Stargardt disease—in childhood also underwent CLIA-certified molecular genetic testing to confirm the presumed or suspected diagnosis of an IRD. Whenever possible, samples from other affected members (ideally both parents when available and, when applicable, also confirmed affected or unaffected siblings) or from the presumed healthy carrier parents in suspected recessive disease were also obtained and tested as part of the diagnostic process to establish the phase of any detected gene mutations and confirm the pathogenicity of detected mutations. All patients underwent as a minimum IRD-focused broad CLIA-certified next-generation sequencing (NGS) testing. The most commonly used labs for diagnostic molecular genetic testing included GeneDx (Gaithersburg, MD) and the Molecular Vision Lab (Hillsboro, OR). Initially negative or inconclusive results generally led also to escalation to deletion/duplication analyses and/or whole exome sequencing (WES) testing when the suspicion of an IRD was especially high. Further details about our approach to molecular genetic testing and diagnostic management of our IRD patients have been recently published (Gupta et al., 2022).

Based on all these criteria, the records of 418 subjects who exhibited positive AAb and/or rIHC test results were reviewed retrospectively. All clinical and imaging findings and test results such as VF and electrophysiological were reviewed. Their correlation with positive AR-AAbs and AON-AAbs and rIHC findings was characterized to infer more precisely the potential pathogenicity of the observed autoreactivities applying criteria as briefly outlined above. When applicable, genetic test results were also reviewed and the presence of any pathogenic, disease-causing genetic changes was confirmed. In addition, we will present more in-depth (but earlier) analyses of our findings in genetic subgroups of patients harboring *EYS* and *USH2A* mutations—these are subsets of genetically well defined patients in which we initially empirically noted an unusually high frequency of inflammatory findings and that have been presented but not published before (Gattegna et al., 2019; Alekseev et al., 2021; Alekseev et al., 2022). These initial observations and our prior presented and published data prompted the more systematic review of our findings that is also presented herein.

## Results

### Autoreactivity patterns in IRD patients by genotype

Of the 418 records reviewed with positive autoreactivities, there were 127 cases (30.3%) with AR-AAbs, AON-AAbs, or both (and/or positive rIHC) who had a molecularly confirmed IRD. All other AAb-positive cases (*n* = 261) had either primary AIR or (more commonly) AINR/ARRON or a paraneoplastic form thereof (CAR/CARON). The latter diagnoses were established based on age and modality of presentation [most often late- and acute/subacute onset, most often asymmetric in nature—i.e., affecting one eye significantly more than the other, which is seldom observed in IRDs except for X-linked RP female carriers (Jacobson et al., 1989)—whether or not in association to a known/confirmed diagnosis of cancer and/or another underlying autoimmune disorder in the affected patient or a family member (Heckenlively and Ferreyra, 2008; Ferreyra et al., 2009; Heckenlively and Lundy, 2018b)] and, in select more ambiguous cases, also based on of inconclusive or outright negative molecular genetic test results. Despite being suspected of having secondary autoimmune complications due to the presence of visible or suspected inflammatory complications based on our selection criteria to warrant AAb and rIHC testing, 9 additional IRD subjects tested negative for any type of AAb and exhibited no staining upon rIHC.

**FIGURE 1:**
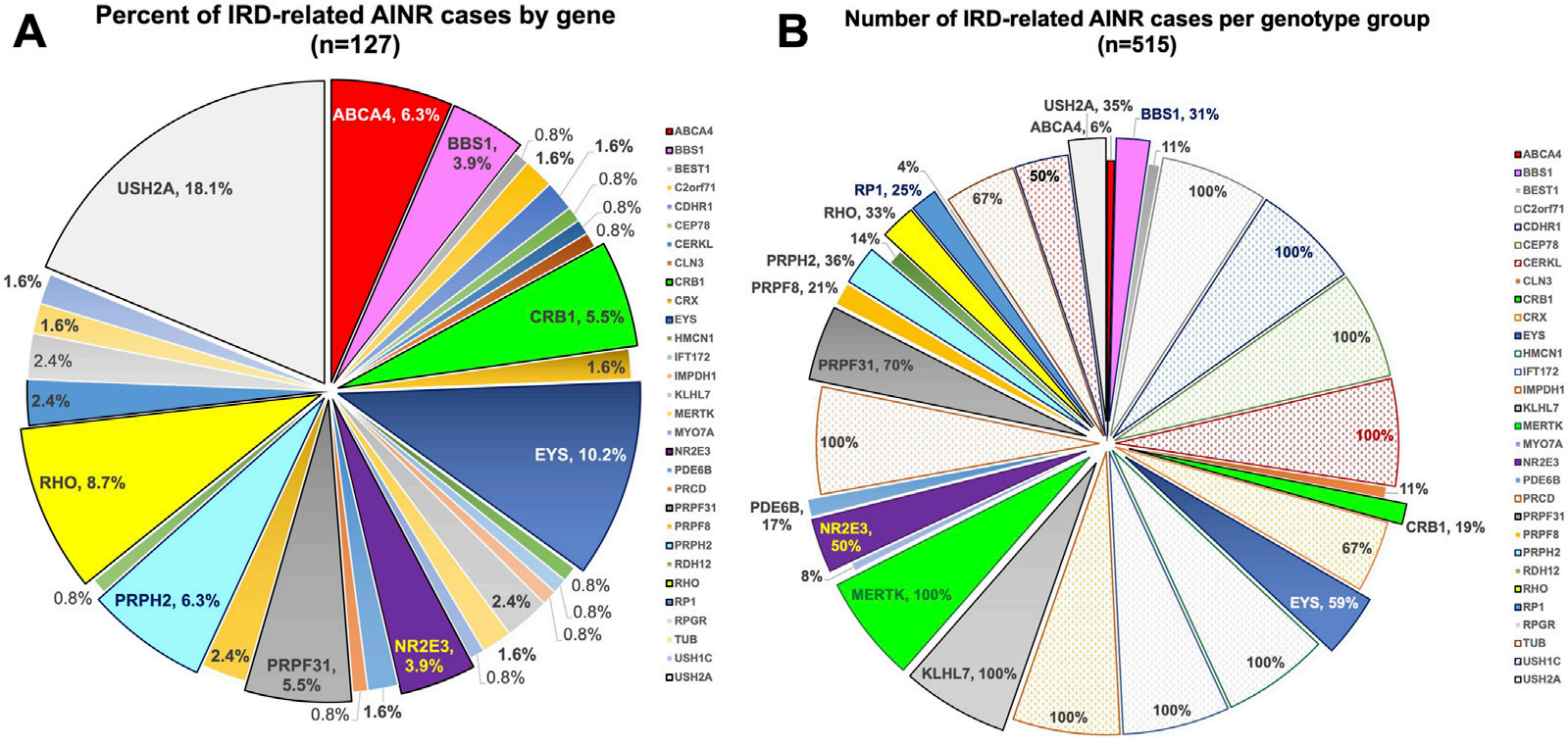
Pie charts illustrating the breakdown by gene of the IRD cases with associated AAb-related inflammatory complications. **(A)** Percentage of cases by gene within the 127 positive IRD cases; **(B)** Percentage of cases within each gene subgroup within our IRD database (*n* = 515 for the genes in which AAbs were observed in 2022). The percentages are cumulative for all AAbs observed, whether only AR-AAbs, only AON-AAbs, or both.

The breakdown of the various genetic etiologies of the 127 IRD cases with positive AR-AAbs, AON-AAbs, or both (and/or positive rIHC) is illustrated in Figure 1A, which illustrates the frequency of the genotypes at play within this data set. To further gain into the prevalence of these positive autoreactivities among all subjects found to have disease-causing in the genes identified by this initial retrospective data analysis, we further reviewed the records of all subjects who, by the end of the data review window period, exhibited mutations in these same genes (*n* = 515). This breakdown is illustrated in Figure 1B and helps us appreciate how, for some rarer genes (such as *MERTK* or *KLHL7*), the within-gene frequency of AAb-associated inflammatory findings was actually very high—in some cases, in as much as 100% of the patients diagnosed with these rarer forms of IRDs. In brief, from this dual-level review of our data, we observed that inflammatory manifestations were especially common among patients with mutations in the *EYS, USH2A, MERTK, CRB1, BBS1, NR2E3, ABCA4, RHO, RP1, KLHL7,* and the *PRPF* family of genes among the latter ones, the *PRPF31* gene more commonly than the *PRPF8* one).

In most of these IRD cases, there was a dual retinal (most often, CME) and optic nerve inflammatory component. Not uncommonly, the latter was a main determinant of the visual acuity and/or field loss above and beyond the CME. A concominant optic neuropathy becomes recognizable not only from measurable RNFL swelling, but also from mismatches between VF areas and extent of ffERG loss. For example, a disproportionate VF loss vis-à-vis far better ffERG responses—with or without asymmetric findings between the two eyes, neither one of which are expected based on published evidence of VF-ERG correlations in IRDs (Iannaccone et al., 1995; Sandberg et al., 1996; Iannaccone et al., 2003)—was a recurrent and particularly telling finding. Other lines of evidence in favor an inflammatory optic nerve component included delayed pattern visual evoked potentials (PVEPs) and/or overt angiographic epi-pr peri-papillary inflammatory findings. In some cases, as it will be illustrated below and as it has been previously reported (Iannaccone and Radic, 2019), the picture could also be associated with a disseminated chorioretinitis-type pattern. Unlike classical uveitis patients, there were typically no anterior chamber cells in IRD patients with secondary AINR, as the process is mostly far posterior. No patient exhibited clear signs of vitritis either, withour instances of vitreal haze. However, it is very important to note that the vast majority of IRD patients affected by a diffuse retinopathy have anterior vitreous cells and, also very commonly, posterior vitreous detachments. These vitreal cells have already been shown to be largely inflammatory in nature (Albert et al., 1986; Newsome and Michels, 1988). A substantial breakdown in the blood-retinal barrier in IRD patients accompanies this process—this is not just clinically intuitive, but it has also been well characterized in the past (Gieser et al., 1980; Fishman and Cunha-Vaz, 1981; Miyake et al., 1984; Travassos et al., 1985; Fishman et al., 1986).

A deeper level analysis of the clinical and functional correlations with AAb reactivity patterns is presented below for the *EYS* and the *USH2A* gene, which are common causes of autosomal recessive RP (ARRP), in the latter case whether with or without hearing loss (Birch et al., 2020; Duncan et al., 2020) and that are also the ongoing object of natural history characterization (NCT03146078, NCT04127006).

### Subgroup analysis of results in ARRP associated with EYS gene mutations

**FIGURE 2:**
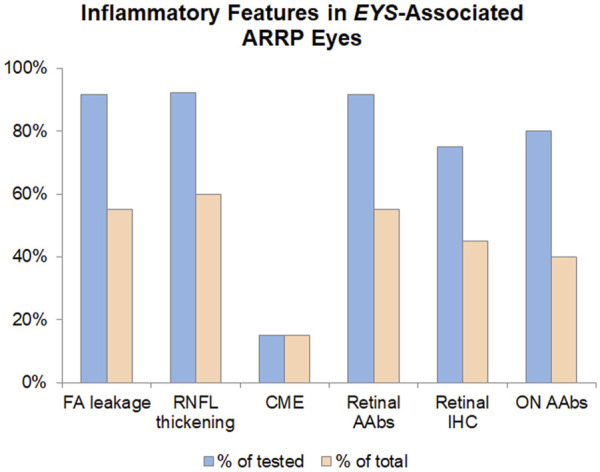
Inflammatory features in *EYS*-Associated ARRP Eyes. The inflammatory component of *EYS*-ARRP was evaluated by leakage on FA (performed in 24 eyes), RNFL thickening on OCT (performed in 26 eyes), and cystoid macular edema on OCT (performed in 40 eyes). Additionally, CLIA-certified testing was performed to detect circulating AAbs against retinal (performed in 12 patients) and retrobulbar optic nerve antigens (performed in 10 patients), as well as retinal immunohistochemistry (performed in 12 patients). Blue bars show positivity as a percentage of tested eyes/patients, whereas orange bars show positivity in the total cohort of 20 *EYS*-ARRP patients.

**FIGURE 3:**
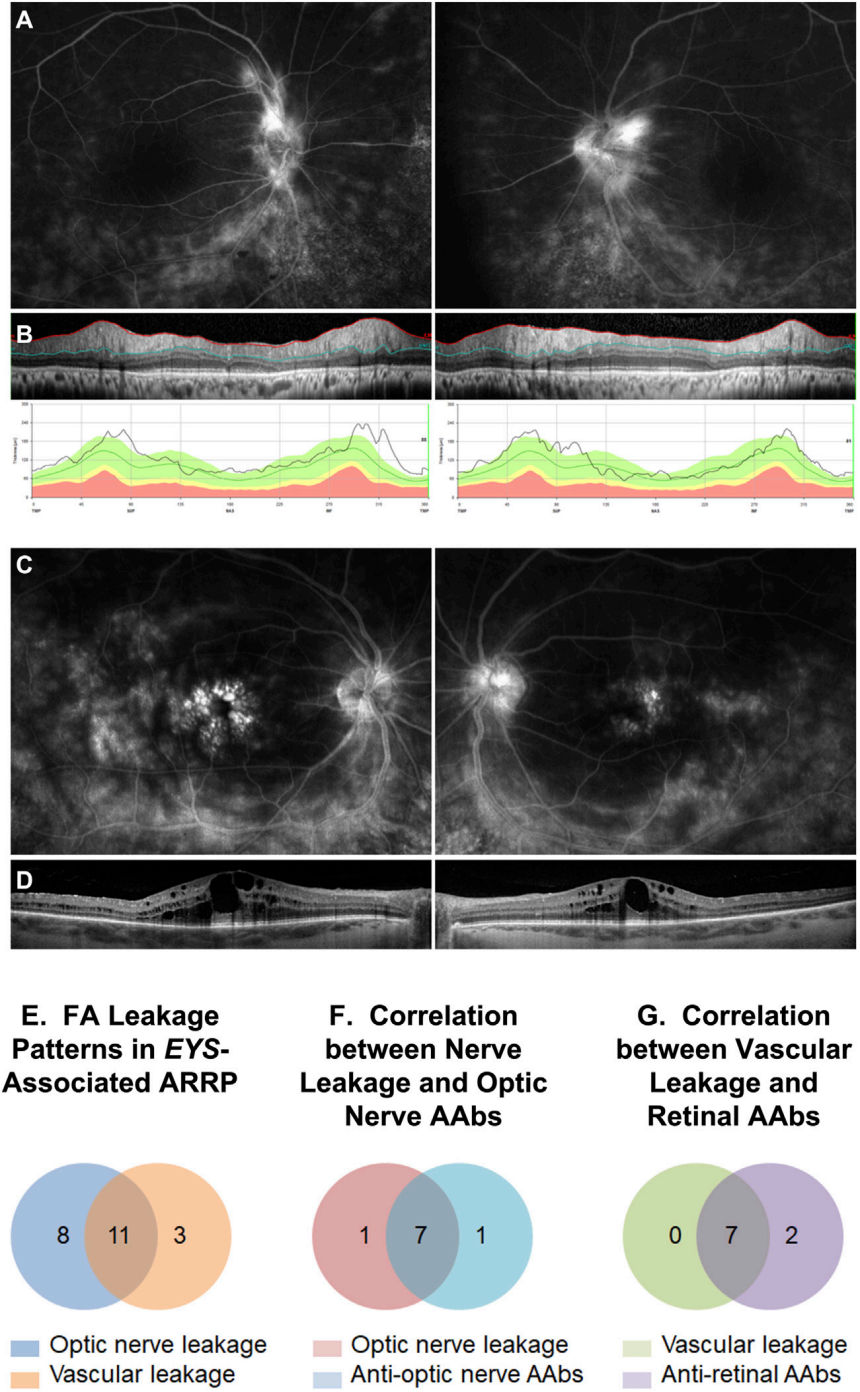
Intravenous fluorescein angiography (IVFA) features in *EYS*-ARRP eyes. Example of optic nerve leakage **(A)** in a patient with sectoral thickening of the retinal nerve fiber layer on disc SD-OCT **(B)**. Example of foveal leakage **(C)** in a patient with CME on macular SD-OCT **(D)**. **(E)** Analysis of IVFA leakage patterns: of 24 analyzed eyes, 19 eyes showed optic nerve leakage, 14 eyes showed perivascular leakage, and 2 eyes showed no leakage. Combined optic nerve and perivascular leakage was seen in 11 eyes. **(F)** Optic nerve leakage on IVFA correlated strongly with presence of circulating AON-AAbs on Western blot. Of the 8 patients who tested positive for the AON-AAbs, 7 showed nerve leakage on FA in at least one eye. Conversely, of the 8 patients with FA nerve leakage, 7 tested positive for AON-AAbs. **(G)** Vascular leakage on IVFA correlated strongly with presence of circulating AR-AAbs. Of the 9 patients who tested positive for AR-AAbs, 7 showed vascular leakage on IVFA in at least one eye. Conversely, all 7 patients with IVFA vascular leakage tested positive for AR-AAbs.

**FIGURE 4:**
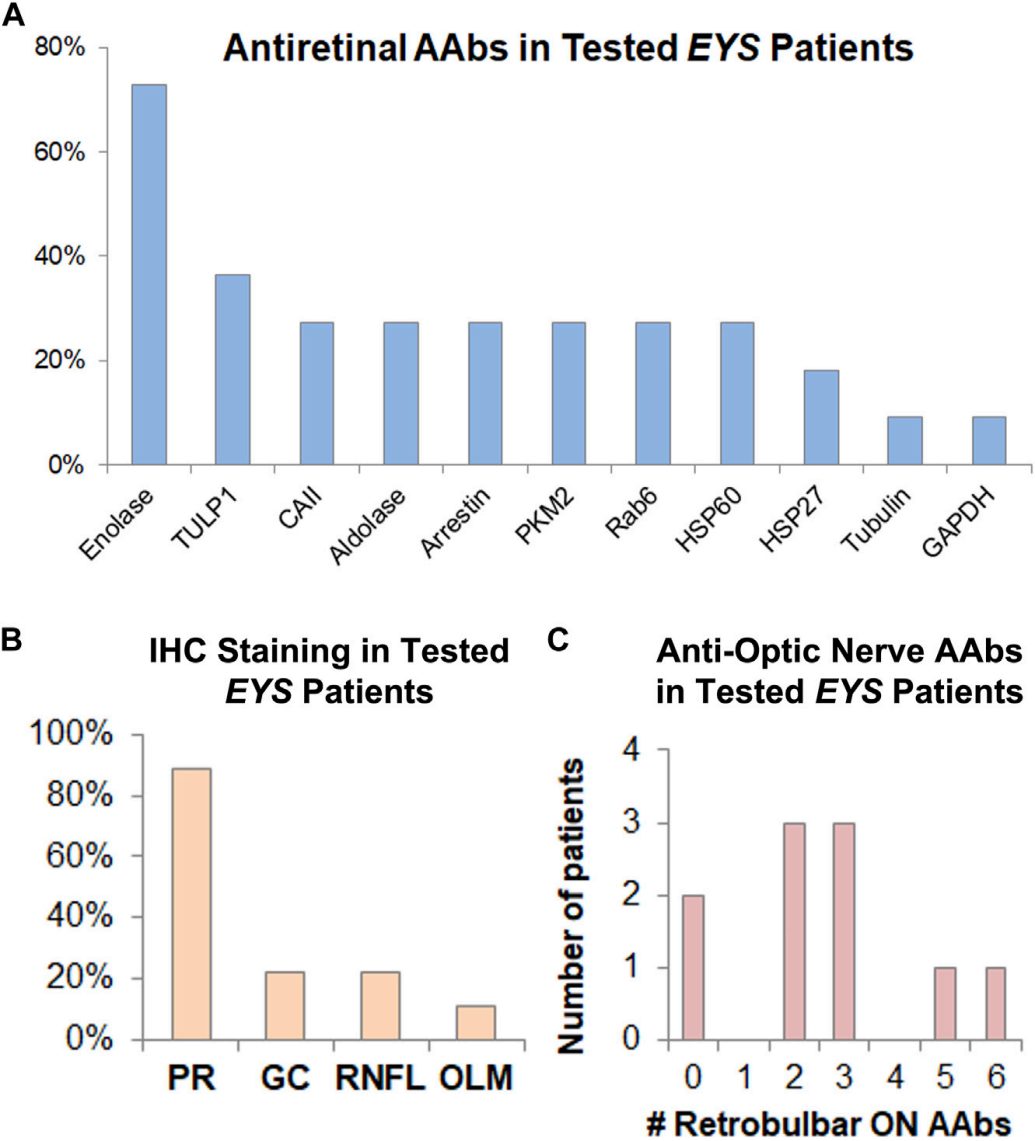
AAbs against retinal and optic nerve antigens are frequently present in *EYS*-ARRP patients. **(A)** Among those patients who tested positive for AR-AAbs, the most prevalent retinal antigen was enolase (involved in 75% of positive patients), followed by TULP1 and others, as shown above. **(B)** Among those patients who had positive tissue staining to rIHC on human retinal tissue sections stained with patient serum, the photoreceptor (PR) layer was the most commonly involved, with less frequent staining of RGCs, RNFL, and outer limiting membrane (OLM). **(C)** Human retrobulbar optic nerve lysates were used for WB-based AON-AAAb testing. The 10 patients who underwent testing, showed positivity, on average, for 2.6 optic nerve antigens, ranging from 0 (no reactivity against retrobulbar antigens) to as many as 6 antigens per patient. Of note, presence of AON-AAbs correlated strongly with optic nerve leakage on IVFA (Figure 3F), and presence of AR-AAbscorrelated strongly with vascular leakage on IVFA (Figure 3G).

In 2021(76), we retrospectively identified 20 subjects positive for biallelic disease-causing *EYS* mutations (M = 8/F = 12, age 23–80), all of whom had undergone a complete eye examination, inclusive of VFs) and ffERGs, macular (*n* = 20) and optic nerve (*n* = 13) SD-OCTs and, in 12 of them, also IVFA and CLIA-certified AAb/rIHC testing. Of the total 24 tested eyes with IVFA, 8 showed optic nerve head leakage, 3 showed leakage at the vascular arcades, and 11 showed combined nerve and vascular leakage. The RNFL was thickened, most often sectorally, in 24 of 26 eyes assessed, and correlated well with IVFA leakage, helping explain disproportionate visual acuity losses compared to foveal findings, or disproportionate VF loss compared to retinal imaging or functional findings. CME was seen by SD-OCT in only 3 of the 20 *EYS* patients, all of them presenting with the latter alone or other associated inflammatory signs. AAbs were identified in all 12 tested subjects. AR-AAbs were found in 11 of the 12 tested patients [most common: against enolase (8/12) and TULP1 (4/12)]. AON-AAbs were found in 8 out of 9 tested patients. AAbs against both tissues were seen in 6 patients, and rIHC showed positive staining in 9 of 12 cases, labeling predominantly photoreceptors (8/12) and less frequently RGCs and the RNFL. The specifics of the observed autoreactivities and related correlations are illustrated in Figure 2, Figure 3, Figure 4.

Altogether, 70% (14/20) of patients exhibited subclinical signs of inflammation and, in 12 of them, they were associated with an autoimmune component that correlated closely with imaging and functional findings. These patients received intravitreal and/or sub-Tenon steroid injections, with both subjective and measurable increases in vision (visual acuity, VFs, or both), associated with improved SD-OCT and IVFA characteristics in most.

### Subgroup analysis of results in USH2A-associated retinal degeneration

In 2022 (Alekseev et al., 2022), we retrospectively identified 75 subjects (M = 38/F = 37, age 4–84) with confirmed disease-causing *USH2A* gene mutations, all of whom had a complete eye exam, including visual acuity (VA), visual fields (VFs), ffERG, macular (*n* = 75) and, in many, optic nerve (*n* = 40) SD-OCT, IVFAs in about 45% of the subjects (*n* = 31), and CLIA-certified testing in 50% (*n* = 35) for circulating AR-AAbsand/or AON-AAbs by antigen-speficic immunoblot, WB, and rIHC.

**FIGURE 5:**
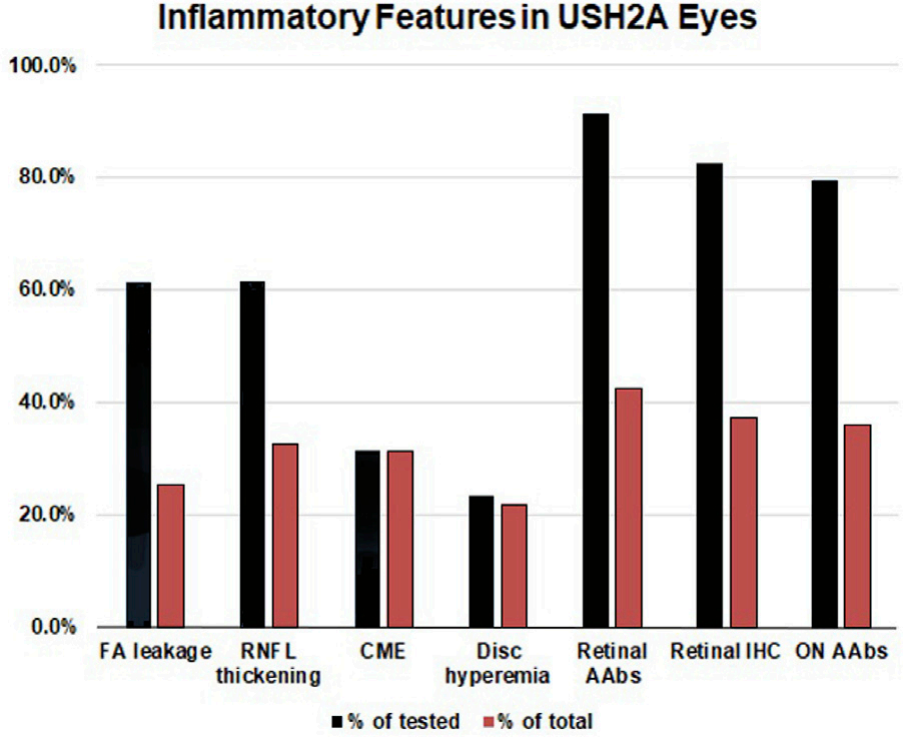
Inflammatory features in USH2A-associated retinal degeneration. The inflammatory component of USH2A-linked ARRP was evaluated by leakage on fluorescein angiography (FA) (*n* = 62 eyes), retinal nerve fiber layer (RNFL) thickening on optical coherence tomography (OCT) (*n* = 80 eyes), cystoid macular edema (CME) on OCT (performed in 150 eyes), and assessment of disc hyperemia on fundoscopy (*n* = 142 eyes). CLIA-certified testing was performed to detect circulating autoantibodies (AAbs) against retinal (*n* = 35) and retrobulbar optic nerve antigens (*n* = 30), as well as retinal immunohistochemistry (IHC, *n* = 34). Blue bars: positivity as a percentage of tested eyes/patients, Red bars: positivity in the total USH2A cohort (*n* = 75).

**FIGURE 6:**
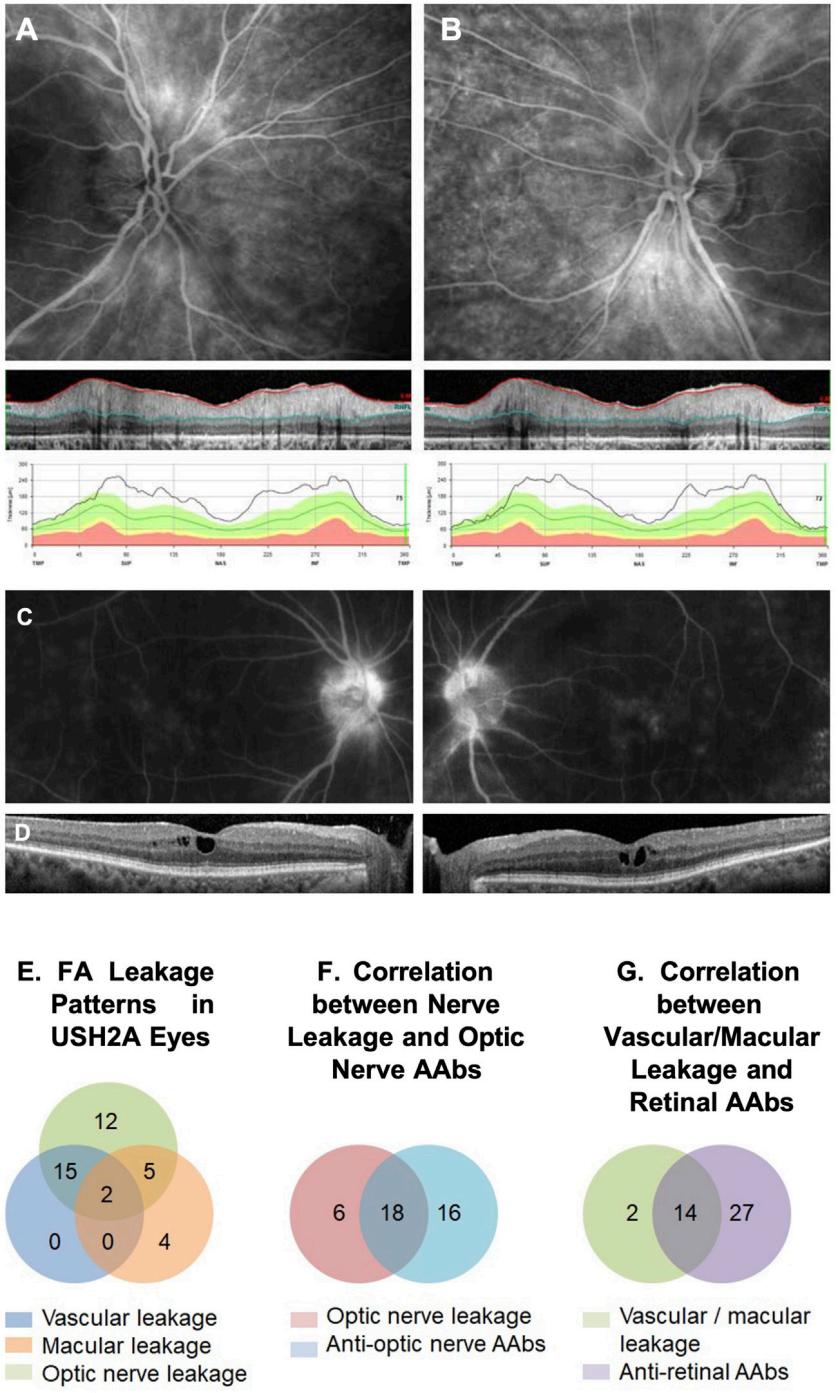
IVFA features and AAb findings in USH2A. An example of a late-phase fluorescein angiography (FA) image of an USH2A-associated retinal degeneration patient shows optic nerve head leakage in both eyes correlated with retinal nerve fiber layer (RNFL) thickening in SD-OCT images **(A, B)**. Similarly, an example of macular leakage on late-phase FA correlated cystoid macular edema (CME) on SD-OCT **(C, D)**. FA leakage pattern in USHA2 eyes showed all ON head, macular, and vascular leakage patterns with overlapping **(E)**. Optic nerve leakage on FA correlated strongly with the presence of circulating anti-optic nerve autoantibodies (ON-AAbs) **(F)**, and Vascular/macular leakage on IVFA correlated strongly with the presence of circulating anti-retinal AAbs (AR-AAbs) **(G)**.

**FIGURE 7:**
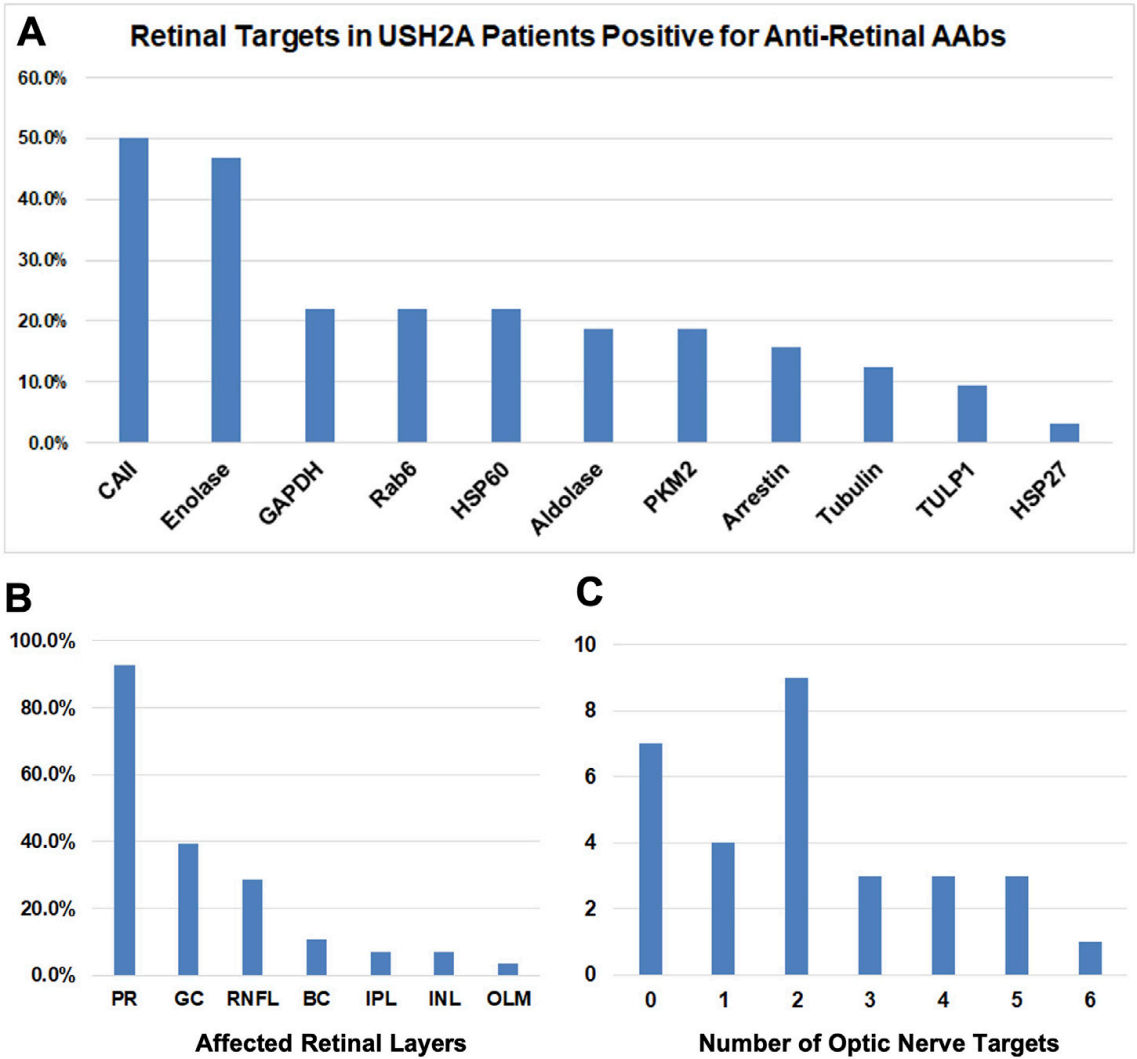
Breakdown of AAbs in USH2A. **(A)** Bar graphs showing results of the retinal targets in USHA 2 patients positive for anti-retinal autoantibodies (AR- AAbs). The presence of autoantibodies against Carbonic anhydrase II and Enolase retinal antigens was demonstrated. **(B)** Bar graphs showing retinal immunohistochemistry (rIHC) results with positive staining predominantly in photoreceptors and less frequently retinal ganglion cells and retinal neural fiber layer. **(C)** Bar graphs showing the number of optic nerve targets.

Of the 62 tested eyes tested with IVFA, 38 eyes had leakage of optic nerve head, vascular arcades, macula, or a combination thereof. The RNFL was thickened on SD-OCT, most often sectorally, in 49 of 80 eyes, and correlated well with IVFA leakage, helping explain disproportionate VA losses compared to foveal SD-OCT findings. CME was seen by SD-OCT in 47 of 150 eyes (31%). AR-AAbswere found in 32 of the 35 tested patients [most often against carbonic anhydrase II (16/35) and enolase (15/35)]. AON-AAbs were found in 28 of 34 tested patients, and rIHC showed positive staining in 28 of 34 cases, labeling predominantly photoreceptors (26/34) and less frequently RGC (11/34) and RNFL (8/34). Altogether, 66.7% (50/75) of patients exhibited subclinical signs of inflammation, which correlated directly with the presence of circulating AAbs in 25 of them. The specifics of the observed autoreactivities and related correlations are illustrated in Figure 5, Figure 6, Figure 7.

Also, these patients, after modest to no response to initial trials of topical and/or oral CAIs for CME and in all cases when significant optic nerve inflammatory involvement was present (which would not be expected to respond to CAIs), received intravitreal and/or sub-Tenon steroid injections in doses and frequencies dictated by the severity of the observed inflammatory findings, with or without subsequent addition of IMT regimens based on response to the intra/periocular steroids. Most patients experienced both subjective and measurable increase in vision (by VA criteria, VF criteria, or both), associated with improved SD-OCT and IVFA characteristics in virtually all treatment-responsive cases.

### Examples of treatment-responsive IRD patients following management of inflammatory complications

How much vision can IRD patients recover from ongoing inflammatory complications? The short answer is—it depends on the severity of the complications at the time of evaluation, the timing of the identification of the complication with respect to any more sudden-onset, abrupt vision changes, and the stage of the underlying disease itself. We have previously briefly reported two cases of IRDs who experienced marked visual loss recovery after management of inflammatory complications associated with detectable AR- and AON-AAbs (Iannaccone and Radic, 2019). One with *RHO*-linked autosomal dominant (AD) RP will be re-reviewed and illustrated in further detail here, alongside a representative case of *EYS*-associated ARRP and even one with *ABCA4-*associated cone-rod dystrophy (CORD). These 3 cases have been specifically chosen to emphasize the very important role of optic nerve inflammatory complications, in IRDs, well beyond CME (if at all present) and how treatment of these cases can allow patients in glaring cases like these ones to achieve their “true retinal potential”.

#### *P23H RHO*-*linked ADRP: Full visual field potential restoration treating inflammatory optic nerve complications*

**FIGURE 8:**
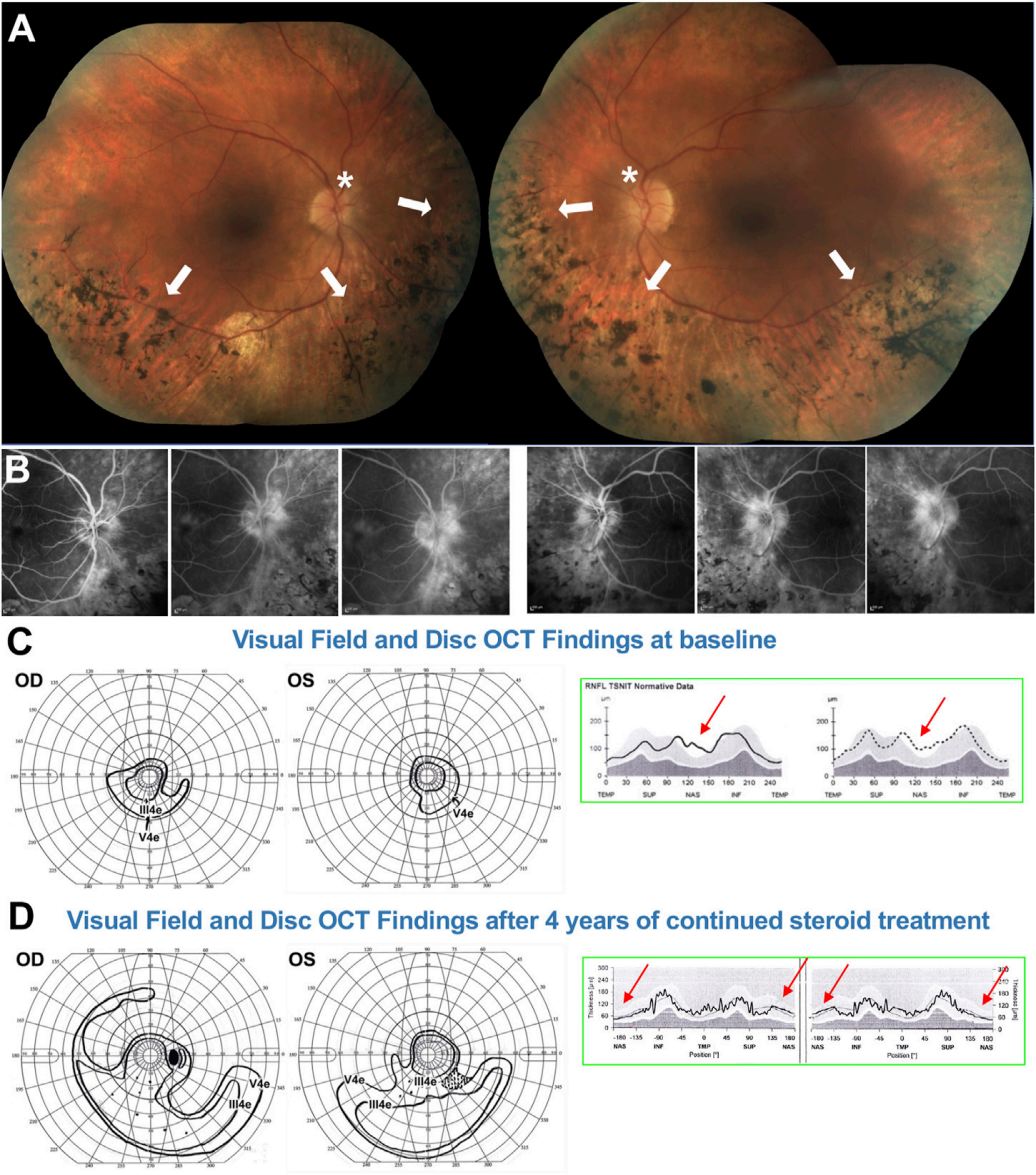
51-yo female with ADRP confirmed to be due to a *RHO* P23H mutation. **(A)** Fundus photos showing altitudinal (inferior) bone spicules (arrows) without disc waxy pallor (asterisk). **(B)** Fluorescein angiography at baseline shows marked papillary and peri-papillary staining and leakage increasing in the later frames. **(C)** left: Baseline kinetic visual fields showing concentric constriction of the visual field, inconsistent with the fundus presentation. **(C)** right: Disc OCT scans show (arrows) nasal RNFL swelling in both eyes. **(D)** left: After treatment, the visual field was markedly improved, with a pattern now consistent with to “true retinal potential” of the patient, and the disc OCT shows resolution of the RNFL swelling in both eyes **(D)** right. Please refer to main text for further details.

This 51-yo White female [briefly reported in (80)] presented with a family history of RP consistent with dominant inheritance. BCVA was 20/20–2 in the right eye and 20/30 in the left. However, the patient complained of blurred vision. Fundus examination showed altitudinal RP limited to the inferior retina (Figure 8A, arrows), a presentation that would predict an altitudinal VF defect. The ffERG (not shown) was reduced but still robust and also rod responses remained partially measurable, attesting to an overall mild to moderate disease stage consistent with the clinical presentation. Of note, the patient’s fundus did not exhibit the typical waxy disc pallor of RP (Figure 8A, asterisks). VF loss was instead disproportionately severe compared to the fundus exam and the ffERG findings, showing a concentric constriction (Figure 8C, left).

Via molecular genetic testing, she was confirmed to harbor an *RHO* P23H mutation. Because of the VF/ffERG and fundus exam mismatch, disc inflammation was also suspected. PVEPs were obtained (not shown), exhibiting marked delays at all tested spatial frequencies (120–140 ms P100 peak times, which is in the optic neuritis range) without amplitude response loss. An IVFA (Figure 8B) showed marked papillary and peri-papillary staining and leakage increasing substantially in the late IVF frames, and peripapillary SD-OCT showed mild but measurable nasal RNFL swelling in both eyes (Figure 8C, right). This suggested a secondary autoimmune component affecting both the optic nerve and retina, causing far more vision loss than caused by the RP alone. The patient serum was positive for anti-ON AAbs against 30-kDa, 35–40-kDa complex, and 46-kDa proteins and for anti-retinal Aabs against 30-kDa (CA-İİ), 33-kDa, 46-kDa (alpha-enolase), 56-, 60-, and 62-kDa proteins, with mild photoreceptor staining and marked RGC and RNFL staining on rIHC. It was therefore decided at this stage to initiate serial subtenon triamcinolone acetonide injections. After 3 years of continued treatment, the disc inflammation and swelling progressively diminished and resolved (Figure 8D, right), and the VF was restituted to her “true retinal potential”, limited to the expected altitudinal VF defect (Figure 8D, left).

#### *EYS*-*linked ARRP: CME resolution and substantial reduction in visual field loss*

**FIGURE 9:**
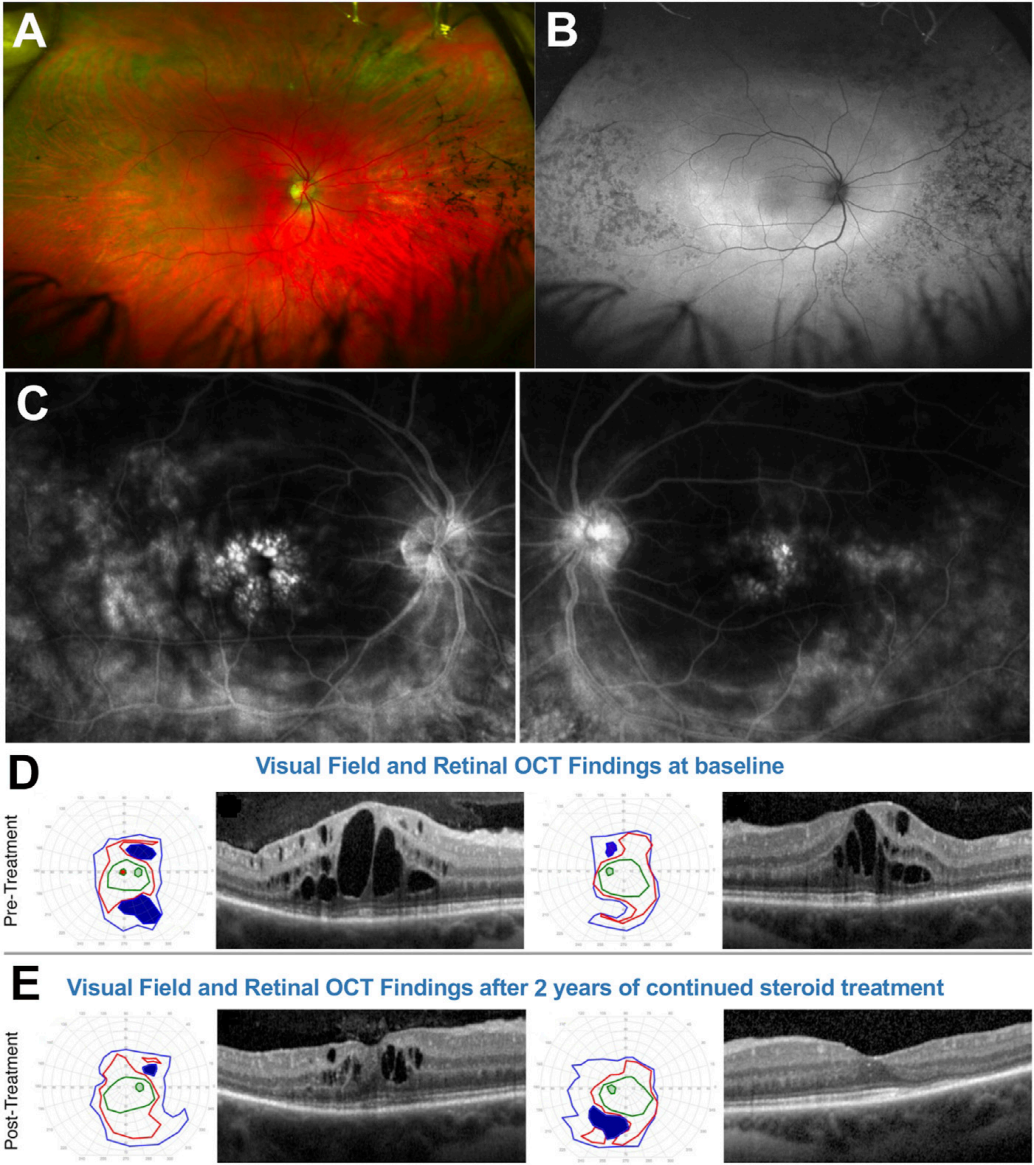
28-yo female with ARRP confirmed to be due to *EYS* mutations. **(A)** Fundus photos. **(B)** Fundus autofluorescence. **(C)** Fluorescein angiography at baseline shows shows marked cystoid macular edema (CME), late optic nerve inflammatory involvement, and hyperfluorescence at the arcades/posterior pole on both eyes. At baseline **(D)** Semiautomated kinetic perimetries (SKPs) show baseline marked bilateral temporal and nasal visual field (VF) loss with other absolute scotomas and macular SD-OCTs also show florid CME. **(E)** After treatment, SKPs show nasal and temporal visual field widening and disappearance of the absolute scotomas seen at baseline. Macular SD-OCTs significantly reduce CME in OD and resolution in OS. Please refer to main text for further details.

This 28yo White female presented with a history of night blindness, VF loss and photopsia that suggested ARRP. Baseline BCVA was 20/50 in the right eye and 20/30 in the left. Her fundus exam (Figure 9A) showed bone spicule-like changes both nasally and, less so, temporally, with only mild nasal/superonasal disc hyperemia bilaterally. These changes were also associated with speckled loss of AF signal in these same areas and a wide ring of hyperautofluorescence was visible at and well outside the vascular arcades, encircling an area of well preserved central retinal integrity (Figure 9B). Genetic testing identified two *EYS* mutations in trans, a c.2259 + 1G>T splice site mutation and c.4103dupT, p. Ser1369IlefsTer18 frameshift mutation. A baseline IVFA (Figure 9C) showed florid CME, which was also apparent on SD-OCT (Figure 9D) and, in addition to this expected finding, it also revealed marked late optic nerve inflammatory involvement and hyperfluorescence at the arcades in both eyes. Baseline SKPs (Figure 9D, far left) showed bilateral temporal and nasal VF loss with other patchy absolute scotomas superiorly and inferiorly in the right eye. There was no response of CME to an initial oral and topical CAI regimen. A secondary autoimmune inflammatory component was suspected, prompting testing for Aabs and rIHC, which identified AON-Aabs against 44, 46, 52, 62, and 136 kDa proteins and for AR-Aabs against 34 k (CRALBP), 46 k (enolase), 52 k (tubulin), 58 k, 58 k (PKM2), and 76 kDa proteins. After adding to the treatment regimen serial bilateral intravitreal triamcinolone acetonide injections, followed by a transition to subtenon injections of the same due to intervening intraocular pressure elevations and oral IMT with long-term MMF, escalated progressively from 500 mg BID to 1,500 mg BID, SKPs at 2 years showed sustained nasal and temporal widening and disappearance of the absolute scotomas seen at baseline with overall improved size and sensitivity, and macular SD-OCTs also showed a significant reduction in CME in the right eye and resolution in the efte ye (Figure 9E). A follow-up IVFA also exhibited markedly reduced leakage at the disc and the arcades (not shown), whereby the improved VF could be attributed to resolved inflammation at the optic nerve and arcade level. These improvements were also associated with a marked reduction in photopsia. After over 5 years of follow up, despite long-term IMT, there have been periodic relapses of the CME, the disc inflammation and portions of the VF loss in conjunction with reductions in the steroid regimen, implemented in an effort to balance this regimen with the IOP status (both oral and topical CAIs have been reintegrated into the regimen to assist with longterm CME and IOP co-management, and a topical NSAID has been added as well), but they all remained susceptible of re-improvement after repeat steroid injections. The current IOP conditions would permit us also to consider transitioning this patient to suprachoroidal triamcinolone acetonide injections at the next CME relapse episode. Because of the precarious IOP status, long-term steroid implants, which would appear to be an ideal solution for this patient, have been deferred for now, but also remain under consideration.

#### *ABCA4*-*linked CORD: From light perception to partial acuity and substantial visual field restitution*

**FIGURE 10:**
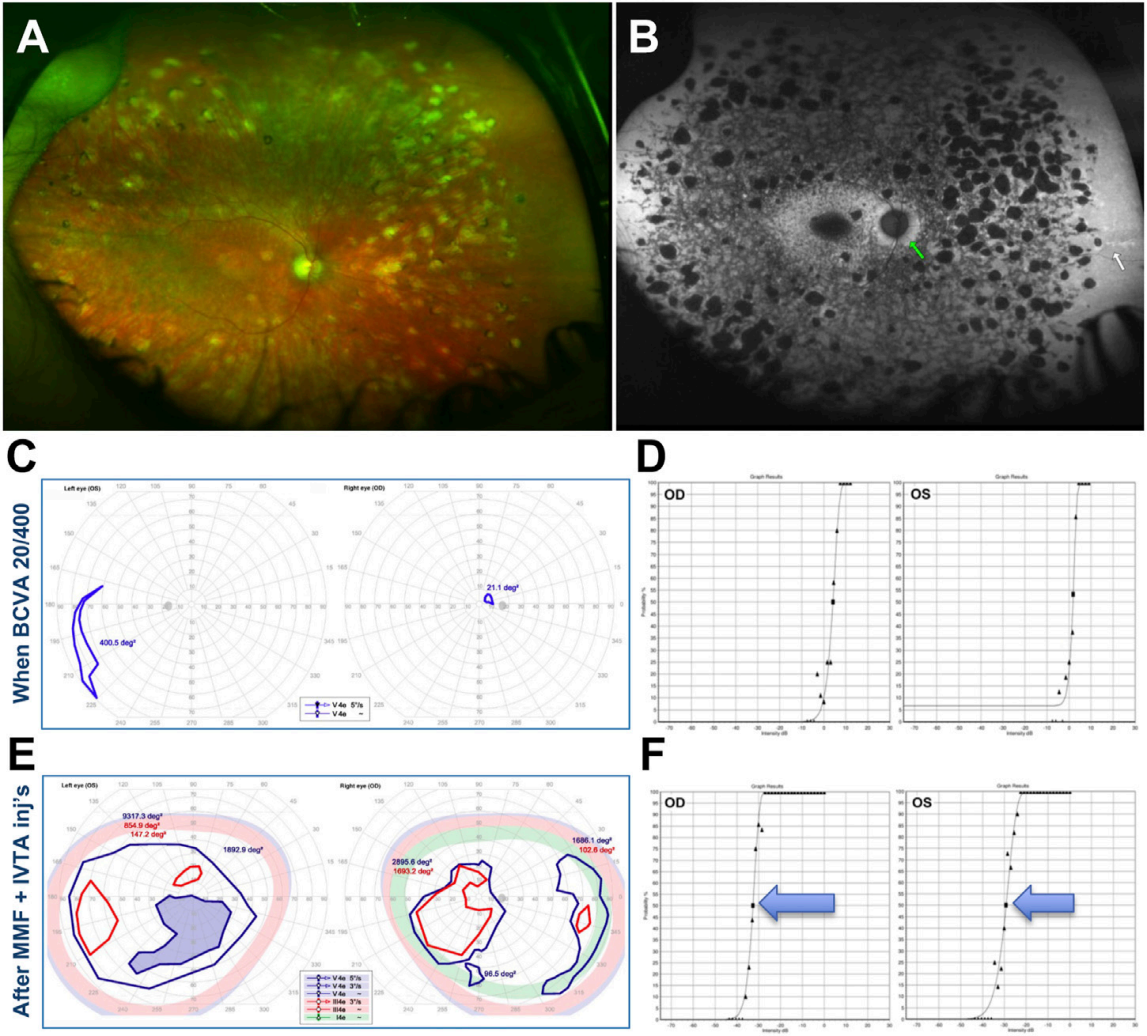
29-year-old White male with confirmed recessive cone-rod dystrophy due to *ABCA4* mutations. **(A)** Fundus photos. **(B)** Fundus autofluorescence. **(C)** Semiautomated kinetic perimetries (SKPs) obtained after the first bout of severe subacute vision loss. **(D)** Full-field Stimulus Threshold (FST) test at this same time point. **(E)** Markedly improved SKPs after treatment. **(F)** Markedly improved FSTs after treatment. Please refer to main text for further details.

A 29-year-old White male with a history of “Stargardt” since childhood presented with a best corrected visual acuity (BCVA) 20/200 with severe loss of ffERG and a widespread phenotype characterized by disseminated atrophic spots, compatible with an advanced stage CORD (Figures 10A, B). The patient was molecularly confirmed to have *ABCA4*-related CORD (2 disease-causing mutations in trans, one of which is a deep intronic c.5461–10T>C​ mutation, which are usually associated with a more severe and progressive phenotype). Typical features of *ABCA4*-related disease such as peripapillary FAF sparing and hyperautofluorescent flecks peripheral to the atrophic spots were noticeable. At this stage, the patient was not subjectively noticing any VF loss and VF was normal to confrontation.

This initial presentation was followed by a sudden, precipitous bilateral BCVA to 20/400 and severe subjective VF loss, which was confirmed by SKP testing (Figure 10C). A Full-Field Stimulus Test (FST) (Birch et al., 2020) obtained at this stage showed severe sensitivity depression to white light stimuli (Figure 10D). At this point, a subacute secondary autoimmune retinal complication was suspected and AAb and rIHC testing was ordered, revealing 4 AR-AAbs (vs. carbonic anhydrase II, aldolase, arrestin, pyruvate kinase M2) and positive rIHC for staining of photoreceptor and RGCs. By the time test results were received and the patient could be seen in follow up, though, BCVA had already declined to light perception (LP) in the right eye and count finger (CF) in the left eye, without cataract or apparent changes in exam or imaging from baseline. A brain MRI had also been obtained to exclude any central origin to the vision loss, and that too was normal. An initial subtenon triamcinolone acetonide injection did not improve the findings, thus MMF 500 mg twice daily and bilateral intravitreal triamcinolone acetonide injections (IVTAs) were started. After the first IVTA injection, BCVA recovered rapidly to the 20/300–20/400 range at distance and 20/70 near, whereby the patient was already able to work successfully at the computer. After serial IVTA injections and continued MMF 500 mg twice daily (18-mo treatment course), BCVA recovered to baseline levels (20/200), VF improved dramatically, and FST testing improved equally dramatically by 3.5–4.5 log units (Figures 10D–F).

## Discussion

We have illustrated the anti-retinal and anti-optic nerve autoreactivity patterns observed in 127 patients reviewed retrospectively with a confirmed molecular genetic diagnosis of an IRD. This patient sample represented 30.3% of the patients with a suspected IRD at referral who tested positive for AAbs. The remainder of the AAb-positive patients were found to haave primary AIR/AINR or its paraneoplastic counterparts (CAR/CARON). Contrary to a common belief that virtually any IRD patient tested will exhibit autoreactivities, we had 9 subjects who, despite being suspected of having secondary autoimmune complications due to the presence of visible or strongly suspected inflammatory complications, tested negative for any type of AAb and exhibited no staining upon rIHC. Thus, despite the sample being upfront more likely to exhibit autoreactivies by virtue of the criteria used to make it medically appropriate to order AAb and rIHC testing, not even everyone in this enriched sample always showed autoreactivity. Our findings show that secondary autoimmune complications in IRDs are indeed common and appear to be more associated with certain genotypes than others. In our retrospective case series, the *EYS, USH2A, MERTK*, *CRB1, BBS1, NR2E3, ABCA4, RHO, RP1, KLHL7,* and the *PRPF* family of genes were among the others more commonly associated with AAb-associated inflammatory manifestations—and among the latter ones, the *PRPF31* gene was more commonly so than the *PRPF8* one. It is not presently known if more immunogenic phenomena associated with certain genotypes may be behind the clustering of AAb autoreactivities within the aforementioned genes. Based on the evidence associated with MERTK gene mutations (reviewed in further detail below), this would be expected to be the case in conjunction with this one gene and may also be the case for the others. This aspect deserves further investigation at the preclinical level. For *BBS1*, the association we found is also quite interesting in light of very recent evidence pointing to this gene, and potentially more in general the BBSome, in being involved in immune synapse assembly by promoting the centrosome polarization to the antigen-presenting cells and in the regulation of selective functions of T cells that predispone BBS patients to other autoimmune and inflammatory diseases (Kanie and Jackson, 2021; Tsyklauri et al., 2021; Stump et al., 2023).

### Evidence for stereotyped pathogenic autoreactivities in MERTK-associated retinal disease

The work of Adamus et al. (Xiong et al., 2013) on the RCS rats, whose retinal dystrophy is due to a mutation in the Mertk gene, has clearly shown how AR-AAbs [directed against arrestin and interphotoreceptor binding protein (IRBP)] develop early in the course of the RCS rat disease. Adamus et al. (Xiong et al., 2013) also showed intense staining of outer photoreceptor segments, TUNEL-positive apoptotic cells that coincide with AAb production, and intense intraretinal microglial and T-lymphocyte reactivity. The microglial reactivity patterns were different than the physiological ones both by tissue level localization and intensity/patterns of activation (Xiong et al., 2013). In line with our observation that these AAb-associated inflammatory reactions often have an optic nerve and retinal vasculitic component, also RCS rats exhibit marked disc and retinal vascular leakage on IVFA (Adamus et al., 2004). Even more importantly, systemic treatment of RCS rats every other day as of P21 (thus once disease had already started to occur) with an epitope-specific biologic agent blocking the anti-IRBP AAbs retain significantly more outer nuclear layer (ONL) thickness than untreated ones or the ones treated with vehicule alone, and neither vascular nor disc leakage and subclinical signs of inflammation are appreciable any longer after active treatment (Adamus et al., 2004).

Following this evidence, we investigated *Mertk-*deficient mice and we observed the same type of anti-arrestin and anti-IRBP in the sera of these mice (Epstein et al., 2015; Iannaccone and Radic, 2019) and that, upon rIHC, *Mertk-*deficient mouse sera stain intensely also the optic nerve head region (Iannaccone and Radic, 2019), which could explain well the disc leakage seen in RCS rats. We further showed that patients harboring the null *MERTK* mutation, p. R775X (Ksantini et al., 2012), exhibit these very same reactivities, indicating that this specific pattern of AAb response is conserved across species when the *MERTK* gene is involved (Epstein et al., 2015; Iannaccone and Radic, 2019). We then showed also that, in conjunction with this AAb response, in *Mertk-*deficient mice there is abundant intraretinal accumulation of large clumps of strongly citrullinated peptides (Hollingsworth et al., 2015; Iannaccone and Radic, 2019), a biochemical reaction that is well known from the rheumatoid arthritis world to be strongly immunogenic and proinflammatory (Iannaccone and Radic, 2019). In addition, we showed that, when *Mertk-*deficient mice are crossed with mice lacking PAD4, which we have demonstrated to be the main retinal peptidylarginine deiminase that mediates intraretinal citrullination under physiological conditions (Hollingsworth et al., 2017; Hollingsworth et al., 2018), the severity of the retinal degenerative phenotype in *Mertk-*deficient mice is approximately half at corresponding time points compared to the same mice with intact PAD4, free to accumulate large amount of immunogenic and proinflammatory citrullinated peptide aggregates (Iannaccone and Radic, 2019). Thus, reducing what we interpret to be a key trigger to the anti-retinal activation of the immune system and production of AAbs in *MERTK-*related disease in mice appears to have at least as meaningful a treatment impact as anti-IRBP AAb blockage has been shown to be in RCS rats.

The presence of a robust inflammatory component in *Mertk*-associated retinal disease has been recently confirmed independently in full by Mercau et al. (Mercau et al., 2023). Thus, one can readily envision how treatment strategies of this type, especially if early enough in the course of the disease, could prove very impactful on patients as well, and not only for patients with *MERTK-*associated disease.

### Evidence for IMT agents as treatments for IRDs

As mentioned in the introduction section, there is now evidence that some IMTs may exert outright beneficial effects on IRDs *via* interactions with the mechanisms intrinsic to the retinal degenerative process itself. One of them is MMF, the very same IMT agent used to help treat inflammatory complications in 2 of our 3 representative cases described above. MMF is a prodrug of mycophenolic acid (MPA), worldwide approved for medical use, which potently suppresses *de novo* guanine nucleotide production by reversibly inhibiting inosine monophosphate dehydrogenase (IMPDH). Yang et al. have discovered that intraperitoneally administered MMF suppresses retinal cyclic guanosine monophosphate (cGMP)-dependent photoreceptor toxicity in both the *rd1* and *rd10* ARRP mouse models, both caused by *PDE6B* mutations (Yang et al., 2020). After treatment, the authors observed marked improvement in retinal microanatomical and functional preservation (cone ffERGs). Since cGMP dysregulation may be a common mechanism of photoreceptor cell death in up to 30% of forms of RP, pursuing additional studies to investigate further the IRD cGMP-dependent mechanism-specific effects of MMF is ongoing. Of further interest, though, MMF is known to be neuroprotective after excitatory injury, to inhibit microglia activation, to inhibit leukocytes, and even act as an antioxidant [reviewed in (90)]. Accordingly, in the *rd1* and *rd10* mouse models treated with MMF, Yang et al. also observed much improved (more physiological state) microglial morphological and migration patterns, and far less photoreceptor layer infiltration by microglial cells. While microglial activation may be important to remove debris resulting from inflammatory events, they also trigger a cascade of inflammatory events, as it has been shown in the *Mertk* murine models discussed above that are connected both directly and indirectly to their migration to non-physiological retinal locations. Thus, notwithstanding the noted cGMP-related effects that make MMF and derivatives thereof attractive as an outright treatment candidate for various forms of RP, a benefit to the inflammatory side of IRDs through its impact on local intraretinal inflammation pathways provides an important validation for its observed clinical benefits in IRD patients with inflammatory, autoimmune-associated complications.

MTX is a drug already routinely used to treat cancer, rheumatoid, psoriatic and other forms of inflammatory arthritis, posterior non-infectious inflammatory eye disease and, intravitreally, ocular lymphoma. The anti-inflammatory actions of MTX encompass multiple mechanisms, including an antimetabolite effect *via* the inhibition of purine and pyrimidine synthesis, transmethylation reactions, translocation of nuclear factor-κB to the nucleus, signaling *via* the Janus kinase–signal transducer and activator of transcription (JAK–STAT) pathway and nitric oxide production, as well as the promotion of adenosine release and expression of certain long non-coding RNAs (Cronstein and Aune, 2020). Like MMF, MTX has been shown to have important, mechanistically specific efficacy in another model of RP, the P23H *RHO* knock-in mouse model (Liu et al., 2020), the same mutation affecting one of the patients we illustrated. Via an *in vitro* screen of hundreds of compounds, Liu et al. discovered that MTX increases the clearance of misfolded P23H *via* the lysosomal pathway and increases also autophagy flux. Intravitreal injections (IVIs) of MTX were then tested *in vitro* in the P23H *RHO* knock-in mouse model for potential therapeutic efficacy at stages when rods were still sufficiently intact to respond to treatment. A regimen of four weekly 25 pmol IVIs started at post-natal day 15 increased misfolded P23H clearance *via* the lysosomal pathway and autophagy also *in vivo*, and led to significantly better functional (ffERG), increased outer segment rhodopsin levels and better ONL preservation compared to untreated mice. A single IVI was insufficient to attain these results, and the 25 pmol dosing outperformed 100 pmol, indicating that there is an optimal therapeutic range for MTX IVIs. Since MTX is a widely available drug and worldwide approved for systemic medical use, and is also available as for IVI regimens in humans, Aldeyra Therapeutics has initiated a human clinical trial (NCT05392179) of IVIs of MTX in ADRP patients with P23H *RHO* mutations and other such mutations in which rhodopsin misfolding and aggregation has already been shown to occur (Iannaccone et al., 2006; Mitchell et al., 2019). Liu et al. did not investigate the behavior of microglial cells in the P23H *RHO* knock-in mice treated with MTX IVIs, thus is it uncertain if, in addition to the noted effects on the clearance of misfolded P23H *via* the lysosomal pathway and the improved autophagy flux, MTX IVIs also improved any inflammatory manifestations that may be at play in this murine model—along the same lines of the case we illustrated. If it did, it too, like MMF, could offer a dual benefit to RP patients and this approach may prove beneficial to other IRD patients who also exhibit inflammatory, autoimmune-mediated complications regardless of any specific effects on the misfolding of accumulating proteins at the intracellular level. More research is needed to this effect and appears worth pursuing—and the outcome of the Aldeyra trial will be of definite interest.

### Limitations and take-home messages from our clinical investigation into the pathogenic potential of AAb-associated inflammation in IRDs

One important limitation of our investigation is its retrospective nature. A prospective investigation would certainly be beneficial. In addition, not all of our IRD patients were tested for AAbs/rIHC, as this can only be done in the setting of a research study and not one where the decision to test for AAbs/rIHC is driven by the presence or suspected presence of inflammation—and, thus, by a criterion of medical necessity. Therefore, the actual prevalence of positive AAbs and IHC tests in IRD patients is not presently known. Furthermore, the mechanism underlying AAb-related vision loss in positive patients with IRDs remains to be elucidated. On the other hand, the pathogenic potential of the same type of AAbs is known from the AIR (Fox et al., 2016; Adamus, 2018) and CAR literature (Adamus et al., 1997; Adamus et al., 1998a; Adamus et al., 1998b; Adamus et al., 2011; Kyger et al., 2013; Xiong et al., 2013). While the exact epitopes involved in the processes may admittedly vary between IRDs, AIR and CAR, one can make the parsimonious assumption that, in the presence of treatable inflammation with tangible benefits associated with these AAbs in IRDs, the pathogenic potential of the AAbs observed in AIR/CAR patients is likely at play also in IRD subjects. Further investigations will be needed to elucidate these nuances.

An important take home message from our experience is that IRD patients with secondary autoimmune complications can, indeed, truly benefit from treatment. As the examples presented herein illustrate well, when the component is impacting primarily the optic nerves, treatment is aimed at letting patients achieve their “full retinal potential” and can be especially impactful. When the secondary autoimmune component affects also the retina beyond mere CME, such as retinal vasculitis or disseminated chorioretinitis-like features, like in the CORD case we presented, treatment can be fundamentally prognosis-altering. Based on these observations, autoimmunity in IRDs cannot be considered just an epiphenomenon, but appears to be an event of pathogenic and prognostic relevance, and of substantial therapeutic importance, a conclusion that is in line with the framework already proposed by Adamus (Adamus, 2021).

We did not perform a case-by-case assessment of the individual therapeutic benefits in our large case series yet, and this will be the object of future retrospective evaluations. However, we have observed how the few patients who would typically exhibit the most modest benefits from steroidal treatment were typically those with more severe, long-lasting vision loss, more severe retinal and optic nerve damage by SD-OCT criteria [loss of central ellipsoid zone (EZ) and external (or outer) limiting membrane (ELM/OLM) or RNFL loss, especially if affecting the papillo-macular bundle (PMB)] and more modest IVFA findings. Most of these patients reported, in their prior history, slow progression of their vision loss over the years until they experienced a sudden acceleration and relatively rapid vision loss that could not be otherwise explained by previous treating physicians. This suggests in these poorly responsive cases that the autoreactivities, at these “burnt-out” stages, truly reflected biomarkers of likely *past* inflammatory autoimmune-mediated manifestations vis-à-vis low levels of ongoing, active inflammation—or, when the IVFA findings were still significant, simply too much preexisting retinal and/or optic nerve damage to respond any further to treatment, emphasizing the likely importance of early detection and treatment of these inflammatory complications.

A common account reported by IRD patients exhibiting autorectivities was also the exacerbation of vision loss within months of undergoing cataract surgery, performed elsewhere, whereby some patients had subsequently refused to have the fellow eye operated on out of fear of experiencing the same outcome. It has been recently reported that CME, the most frequent post-operative inflammatory complication of cataract surgery (also known as the Irvine-Gass syndrome) is much more common in RP patients than in the general population (Hong et al., 2020; Antonio-Aguirre et al., 2022; Nguyen et al., 2023). There is no accounting in the literature for the optic nerve inflammatory complications in IRDs. However, over the past 6+ years, it has been our experience that the risk for post-cataract inflammatory complications can be minimized in IRD patients. Due to our heightened level of awareness of these issues, we instituted early on a systematic protocol whereby no IRD patient undergoes cataract surgery at our facility until examined by the IRD specialist and confirmed to have minimal to no underlying inflammatory findings. When present, AAb testing has been obtained and, if positive, surgeries have been strategically delayed until inflammation has been subdued. To date, contrary to what has been reported in the literature (Hong et al., 2020; Antonio-Aguirre et al., 2022; Nguyen et al., 2023), among hundreds of IRD patients who have undergone cataract surgery at our facility since 2016, we have observed less than a handful of cases of minimal and rapidly resolved post-operative CME, having had each of these “inflammation-prone” IRD cases pretreated shortly before cataract surgery with—usually posterior subtenon—steroids, typically within a week of surgery. None of these patients, even after years of follow up, has to date suffered any sudden or major disease progression or vision loss after surgery not even in the patients who had experienced as much no-light-perception outcomes in the fellow eye operated on previously elsewhere before the autoimmune-mediated inflammatory substrate had been recognized and managed. We aim to conduct a detailed retrospective review of cataract surgery-specific outcomes in our IRD patient population pretreated with our protocol in a separate setting, since this is not the focus of this manuscript. However, we wanted to emphasize herein the substantial practical relevance of this observation to outcomes and prognosis for IRD patients who so often need to undergo cataract surgery and, otherwise, tend to benefit greatly from cataract removal (Hong et al., 2020; Antonio-Aguirre et al., 2022; Nguyen et al., 2023). We have found this to be yet another very important and immediately relevant ramification of our findings that has impactful implications when it comes to ensuring that we do everything possible to avoid accidentally contributing closing the door on the light that *is* at the end of the tunnel for IRDs. We sincerely hope that, having shared this experience, the same caution and prudence in preevaluating (and pretreating, if need be) IRD patients that we use ahead of cataract surgery will be used by everyone henceforth.

## Conclusion

In summary, AAb-associated inflammation in IRDs is not a mere epiphenomenon and, at least in many cases, it is truly impactful yet treatable, in line with the mechanistic framework already proposed by Adamus (Adamus, 2021). Thus, while this double-edged sword can prove to be a significant worsening factor for the prognosis of affected patients, recognizing the presence of inflammation in IRDs and treating it can be truly beneficial. The benefits are multiple: a) inflammation can be a potentially strong confounder in natural history studies, if not accounted for; since inflammation appears to be part of the natural history of IRDs, assessing patients for these issues should be incorporated in natural history studies, and should not lead to excluding patients who have such complications; b) inflammation can impact adversely safety and efficacy outcomes in gene therapy trial efforts and other gene-agnostic treatments (e.g., optogenetics, stem cell-related applications); c) successful treatment of inflammatory complications in IRDs can significantly expand the viable therapeutic window for many IRD patients, thereby making them more likely to ultimately remain eligible for gene-specific and agnostic treatment approaches—some of which could, in a not too distant future, include also approaches to better management of intraretinal inflammation including use of existing IMTs or as, but by no means not limited to, PAD4 blockage/inhibition as we showed potentially possible in *Mertk-*deficient mice (Iannaccone and Radic, 2019). Ultimately, one may envision a future in which one may also be able to recognize, perhaps with the aid of artificial intelligence and machine learning-based approaches, patterns of better response to treatment associated with certain pharmacological regimens and, perhaps, certain AAb profiles, allowing us also to refine the management of inflammation in IRDs, identify optimal treatment protocols, and achieve a better level of truly personalized medicine-type approaches also for IRDs.

## Data Availability

The datasets presented in this article are not readily available because There are no datasets that can be made available for this investigation because the data cannot be made publicly available for both medicolegal and ethical reasons related to HIPAA regulations protecting access to personal health information, or PHI. Requests to access the datasets should be directed to aiannacc@yahoo.com.

## References

[B1] Adamus G. Amundson D. Seigel G. Machnicki M. (1998). Anti-enolase-alpha autoantibodies in cancer-associated retinopathy: Epitope mapping and cytotoxicity on retinal cells. J. Autoimmun. 11 (6), 671–677. 10.1006/jaut.1998.0239 9878089

[B2] Adamus G. (2018). Are anti-retinal autoantibodies a cause or a consequence of retinal degeneration in autoimmune retinopathies? Front. Immunol. 9, 765. 10.3389/fimmu.2018.00765 29713325 PMC5911469

[B3] Adamus G. Brown L. Schiffman J. Iannaccone A. (2011). Diversity in autoimmunity against retinal, neuronal, and axonal antigens in acquired neuro-retinopathy. J. Ophthalmic Inflamm. Infect. 1 (3), 111–121. 10.1007/s12348-011-0028-8 21744285 PMC3168374

[B4] Adamus G. (2020). Current techniques to accurately measure anti-retinal autoantibodies. Expert Rev. Ophthalmol. 15 (2), 111–118. 10.1080/17469899.2020.1739522 32318114 PMC7172387

[B5] Adamus G. (2021). Importance of autoimmune responses in progression of retinal degeneration initiated by gene mutations. Front. Med. (Lausanne) 8, 672444. 10.3389/fmed.2021.672444 34926479 PMC8674421

[B6] Adamus G. Machnicki M. Elerding H. Sugden B. Blocker Y. S. Fox D. A. (1998). Antibodies to recoverin induce apoptosis of photoreceptor and bipolar cells*in vivo* . J. Autoimmun. 11 (5), 523–533. 10.1006/jaut.1998.0221 9802939

[B7] Adamus G. Machnicki M. Seigel G. M. (1997). Apoptotic retinal cell death induced by antirecoverin autoantibodies of cancer-associated retinopathy. Investigative Ophthalmol. Vis. Sci. 38 (2), 283–291. 9040460

[B8] Adamus G. Ren G. Weleber R. G. (2004). Autoantibodies against retinal proteins in paraneoplastic and autoimmune retinopathy. BMC Ophthalmol. 4, 5. 10.1186/1471-2415-4-5 15180904 PMC446200

[B9] Adamus G. Wang S. Kyger M. Worley A. Lu B. Burrows G. G. (2012). Systemic immunotherapy delays photoreceptor cell loss and prevents vascular pathology in Royal College of Surgeons rats. Mol. Vis. 18, 2323–2337. 22977300 PMC3441155

[B10] Ahn S. J. Kim K. E. Woo S. J. Park K. H. (2014). The effect of an intravitreal dexamethasone implant for cystoid macular edema in retinitis pigmentosa: A case report and literature review. Ophthalmic Surg. Lasers Imaging Retina 45 (2), 160–164. 10.3928/23258160-20140131-03 24506098

[B11] Albert D. M. Pruett R. C. Craft J. L. (1986). Transmission electron microscopic observations of vitreous abnormalities in retinitis pigmentosa. Am. J. Ophthalmol. 101 (6), 665–672. 10.1016/0002-9394(86)90766-x 3717249

[B12] Alekseev O. Adamus G. Iannaccone A. (2021). Inflammatory findings in autosomal recessive retinitis pigmentosa (ARRP) associated with EYS gene mutations. Invest. Ophthalmol. Vis. Sci. 62 (7), 3234.

[B13] Alekseev O. Krauss E. Kedrov M. Adamus G. Iannaccone A. (2022). Retinal and optic nerve inflammatory findings are a common feature in patients with USH2A-associated retinal degeneration. Invest. Ophthalmol. Vis. Sci. 63 (7), 4498.

[B14] Androudi S. Letko E. Meniconi M. Papadaki T. Ahmed M. Foster C. S. (2005). Safety and efficacy of intravitreal triamcinolone acetonide for uveitic macular edema. Ocul. Immunol. Inflamm. 13 (2-3), 205–212. 10.1080/09273940590933511 16019680

[B15] Ansari A. S. Amir Z. Williams G. S. (2021). Bilateral 0.19 mg fluocinolone acetonide intravitreal implant in the successful treatment of juvenile idiopathic arthritis-associated uveitis and secondary macular oedema: A case report and review of intravitreal therapies. Ophthalmol. Ther. 10 (1), 193–200. 10.1007/s40123-020-00328-9 33464558 PMC7887104

[B16] Antonio-Aguirre B. Swenor B. Canner J. K. Singh M. S. (2022). Risk of cystoid macular edema after cataract surgery in retinitis pigmentosa: An analysis of United States claims from 2010 to 2018. Ophthalmol. Retina 6 (10), 906–913. 10.1016/j.oret.2022.04.018 35513237

[B17] Apushkin M. A. Fishman G. A. Grover S. Janowicz M. J. (2007). Rebound of cystoid macular edema with continued use of acetazolamide in patients with retinitis pigmentosa. Retina 27 (8), 1112–1118. 10.1097/IAE.0b013e31805f6b79 18040255

[B18] Asproudis I. Katsanos A. Kozeis N. Tantou A. Konstas A. G. (2017). Update on the treatment of uveitis in patients with juvenile idiopathic arthritis: A review. Adv. Ther. 34 (12), 2558–2565. 10.1007/s12325-017-0635-3 29143927

[B19] Bakthavatchalam M. Lai F. H. P. Rong S. S. Ng D. S. Brelen M. E. (2018). Treatment of cystoid macular edema secondary to retinitis pigmentosa: A systematic review. Surv. Ophthalmol. 63 (3), 329–339. 10.1016/j.survophthal.2017.09.009 28987613

[B20] Berson E. L. Sandberg M. A. Rosner B. Birch D. G. Hanson A. H. (1985). Natural course of retinitis pigmentosa over a three-year interval. Am. J. Ophthalmol. 99, 240–251. 10.1016/0002-9394(85)90351-4 3976802

[B21] Birch D. G. Cheng P. Duncan J. L. Ayala A. R. Maguire M. G. Audo I. (2020). The RUSH2A study: Best-corrected visual acuity, full-field electroretinography amplitudes, and full-field Stimulus thresholds at baseline. Transl. Vis. Sci. Technol. 9 (11), 9. 10.1167/tvst.9.11.9 PMC7552938 33133772

[B22] Bourgault S. Aroichane M. Wittenberg L. A. Lavallee A. Ma P. E. (2013). Treatment of refractory uveitic macular edema with dexamethasone intravitreal implants in a pediatric patient with bilateral granulomatous idiopathic panuveitis: A case report. J. Ophthalmic Inflamm. Infect. 3 (1), 61. 10.1186/1869-5760-3-61 24148192 PMC4016568

[B23] Brinkman C. Pinckers A. Broekhuyse R. (1980). Immune reactivity to different retinal antigens in patients suffering from retinitis pigmentosa. Investigative Ophthalmol. Vis. Sci. 19 (7), 743–750. 6993414

[B24] Broekhuyse R. Van Herck M. Pinckers A. Winkens H. Van Vugt A. Ryckaert S. (1988). Immune responsiveness to retinal S-antigen and opsin in serpiginous choroiditis and other retinal diseases. Doc. Ophthalmol. 69 (1), 83–93. 10.1007/BF00154420 2971518

[B25] Chan C. C. Hooks J. J. Nussenblatt R. B. Detrick B. (1986). Expression of Ia antigen on retinal pigment epithelium in experimental autoimmune uveoretinitis. Curr. Eye Res. 5 (4), 325–330. 10.3109/02713688609020059 3486745

[B26] Chant S. M. Heckenlively J. Meyers-Elliott R. H. (1985). Autoimmunity in hereditary retinal degeneration. I. Basic studies. Br. J. Ophthalmol. 69 (1), 19–24. 10.1136/bjo.69.1.19 3880639 PMC1040515

[B27] Chen C. Liu X. Peng X. (2022). Management of cystoid macular edema in retinitis pigmentosa: A systematic review and meta-analysis. Front. Med. (Lausanne). 9, 895208. 10.3389/fmed.2022.895208 35652079 PMC9149278

[B28] Chronopoulos A. Chronopoulos P. Hattenbach L. O. Ashurov A. Schutz J. S. Pfeiffer N. (2022). Intravitreal fluocinolone acetonide implant for chronic postoperative cystoid macular edema - two years results. Eur. J. Ophthalmol. 33, 1054–1060. 10.1177/11206721221124688 36062617

[B29] Cideciyan A. V. Zhao X. Nielson L. Khani S. C. Jacobson S. G. Palczewski K. (1998). Null mutation in the rhodopsin kinase gene slows recovery kinetics of rod and cone phototransduction in man. Proc. Natl. Acad. Sci. U. S. A. 95, 328–333. 10.1073/pnas.95.1.328 9419375 PMC18214

[B30] Cronstein B. N. Aune T. M. (2020). Methotrexate and its mechanisms of action in inflammatory arthritis. Nat. Rev. Rheumatol. 16 (3), 145–154. 10.1038/s41584-020-0373-9 32066940

[B31] Daiger S. (2020). Summaries of genes and loci causing retinal diseases (RetNet). Houston, TX, USA: The University of Texas Health Science Center.

[B32] Davoudi S. Ebrahimiadib N. Yasa C. Sevgi D. D. Roohipoor R. Papavasilieou E. (2017). Outcomes in autoimmune retinopathy patients treated with rituximab. Am. J. Ophthalmol. 180, 124–132. 10.1016/j.ajo.2017.04.019 28483493

[B33] Detrick B. Newsome D. A. Percopo C. M. Hooks J. J. (1985). Class II antigen expression and gamma interferon modulation of monocytes and retinal pigment epithelial cells from patients with retinitis pigmentosa. Clin. Immunol. Immunopathol. 36 (2), 201–211. 10.1016/0090-1229(85)90121-7 3924457

[B34] Detrick B. Rodrigues M. Chan C. C. Tso M. O. Hooks J. J. (1986). Expression of HLA-DR antigen on retinal pigment epithelial cells in retinitis pigmentosa. Am. J. Ophthalmol. 101 (5), 584–590. 10.1016/0002-9394(86)90949-9 3518466

[B35] Doycheva D. Deuter C. Grajewski R. (2018). Topical corticosteroids and non-steroidal anti-inflammatory drugs in the therapy of non-infectious uveitis. Klin. Monatsblatter fur Augenheilkd. 235 (5), 586–591. 10.1055/a-0590-4546 29739028

[B36] Duncan J. L. Liang W. Maguire M. G. Audo I. Ayala A. R. Birch D. G. (2020). Baseline visual field findings in the RUSH2A study: Associated factors and correlation with other measures of disease severity. Am. J. Ophthalmol. 219, 87–100. 10.1016/j.ajo.2020.05.024 32446738 PMC8596302

[B37] Epstein R. S. New D. D. Hollingsworth T. J. Meunier I. Lenchik N. I. Lu Q. (2015). Defective mer-tyrosine kinase (mer-TK) function is associated with anti-arrestin and anti-interphotoreceptor retinoid-binding protein (IRBP) autoantibodies (AAbs) in Mer, Axl, Tyro3 -/- (TAM) mice and in autosomal recessive retinitis pigmentosa (arRP) patients with a null MERTK mutation. Invest. Ophthalmol. Vis. Sci. 56. E-Abstract 169.

[B38] Epstein R. S. Sollenberger E. Adamus G. Iannaccone A. (2014). Clinical, functional, and imaging characteristics of cancer-associated retinopathy and optic neuropathy. Chicago, IL: American Academy of Ophthalmology meeting. Oct. 18-22,.

[B39] Feiler D. L. Srivastava S. K. Pichi F. Bena J. Lowder C. Y. (2017). Resolution of noninfectious uveitic cystoid macular edema with topical difluprednate. Retina 37 (5), 844–850. 10.1097/IAE.0000000000001243 27529841

[B40] Ferreyra H. A. Jayasundera T. Khan N. W. He S. Lu Y. Heckenlively J. R. (2009). Management of autoimmune retinopathies with immunosuppression. Arch. Ophthalmol. 127 (4), 390–397. 10.1001/archophthalmol.2009.24 19365013

[B41] Finn A. P. Keenan R. T. Jaffe G. J. (2020). Reconstitution of the ellipsoid zone with tocilizumab in autoimmune retinopathy. Retin. Cases Brief Rep. 14 (4), 297–300. 10.1097/ICB.0000000000000766 29952844

[B42] Fishman G. A. Cunha-Vaz J. E. (1981). Carriers of X-linked recessive retinitis pigmentosa: Investigation by vitreous fluorophotometry. Int. Ophthalmol. 4 (1-2), 37–44. 10.1007/BF00139579 7197669

[B43] Fishman G. A. Rhee A. J. Blair N. P. (1986). Blood-retinal barrier function in patients with cone or cone-rod dystrophy. Arch. Ophthalmol. 104 (4), 545–548. 10.1001/archopht.1986.01050160101022 3954658

[B44] Forte R. Pannarale L. Iannaccone A. Vingolo E. M. Santi G. Pannarale M. R. (1994). Cystoid macular edema in retinitis pigmentosa: Clinical and functional evaluation of patients treated with deflazacort. [ARVO abstracts]. Invest. Ophthalmol. VisSci 35 (4), 1958.

[B45] Fox A. R. Gordon L. K. Heckenlively J. R. Davis J. L. Goldstein D. A. Lowder C. Y. (2016). Consensus on the diagnosis and management of nonparaneoplastic autoimmune retinopathy using a modified delphi approach. Am. J. Ophthalmol. 168, 183–190. 10.1016/j.ajo.2016.05.013 27210277 PMC4969197

[B46] Frere A. Caspers L. Makhoul D. Judice L. Postelmans L. Janssens X. (2017). Single dexamethasone intravitreal implant in the treatment of noninfectious uveitis. J. ocular Pharmacol. Ther. official J. Assoc. Ocular Pharmacol. Ther. 33 (4), 290–297. 10.1089/jop.2016.0139 28448238

[B47] Frishman L. Sustar M. Kremers J. McAnany J. J. Sarossy M. Tzekov R. (2018). ISCEV extended protocol for the photopic negative response (PhNR) of the full-field electroretinogram. Doc. Ophthalmol. 136 (3), 207–211. 10.1007/s10633-018-9638-x 29855761 PMC6061118

[B48] Fuchs S. Nakazawa M. Maw M. Tamai M. Oguchi Y. Gal A. (1995). A homozygous 1-base pair deletion in the arrestin gene is a frequent cause of Oguchi disease in Japanese. Nat. Genet. 10 (3), 360–362. 10.1038/ng0795-360 7670478

[B49] Funatsu J. Murakami Y. Shimokawa S. Nakatake S. Fujiwara K. Okita A. (2022). Circulating inflammatory monocytes oppose microglia and contribute to cone cell death in retinitis pigmentosa. PNAS Nexus 1 (1), pgac003. 10.1093/pnasnexus/pgac003 35529318 PMC9075747

[B50] Gattegna R. Bleicher I. Iannaccone A. (2019). Emerging phenotypic characteristics and identification of novel mutations in autosomal recessive retinitis pigmentosa (ARRP) associated with the EYS gene. Invest. Ophthalmol. Vis. Sci. 60 (7), 4508.

[B51] Gieser D. K. Fishman G. A. Cunha-Vaz J. (1980). X-linked recessive retinitis pigmentosa and vitreous fluorophotometry. A study of female heterozygotes. Arch. Ophthalmol. 98 (2), 307–310. 10.1001/archopht.1980.01020030303013 7352882

[B52] Grewal D. S. Jaffe G. J. Keenan R. T. (2021). Sarilumab for recalcitrant cystoid macular edema in non-paraneoplastic autoimmune retinopathy. Retin. Cases Brief Rep. 15 (5), 504–508. 10.1097/ICB.0000000000000872 30986811 PMC6783341

[B53] Grixti A. Hagan R. Nayak H. Chandna A. (2016). Multifocal choroiditis with panuveitis in an 8-year-old boy with long-standing idiopathic acute anterior uveitis. Eur. J. Ophthalmol. 26 (5), e114–e117. 10.5301/ejo.5000772 26951535

[B54] Grover S. Apushkin M. A. Fishman G. A. (2006). Topical dorzolamide for the treatment of cystoid macular edema in patients with retinitis pigmentosa. Am. J. Ophthalmol. 141 (5), 850–858. 10.1016/j.ajo.2005.12.030 16546110

[B55] Grover S. Fishman G. A. Fiscella R. G. Adelman A. E. (1997). Efficacy of dorzolamide hydrochloride in the management of chronic cystoid macular edema in patients with retinitis pigmentosa. Retina 17 (3), 222–231. 10.1097/00006982-199705000-00009 9196934

[B56] Gupta P. Kheir W. Peng B. Chiang J. P-W. Iannaccone A. (2022). Careful clinical-functional phenotyping combined with systematic, broad NGS Panel-based genotyping identify numerous novel disease-causing mutations and deletions in inherited retinal disease patients. Mol. Vis. 28, 202–217. PMC9514548 36284670

[B57] Hariprasad S. M. Callanan D. (2008). Topical nepafenac 0.1% for treatment of chronic uveitic cystoid macular edema. Retin Cases Brief. Rep. 2 (4), 304–308. 10.1097/ICB.0b013e31809ed9db 25390598

[B58] Hasanreisoglu M. Ozdemir H. B. Ozkan K. Yuksel M. Aktas Z. Atalay H. T. (2019). Intravitreal dexamethasone implant in the treatment of non-infectious uveitis. Turk J. Ophthalmol. 49 (5), 250–257. 10.4274/tjo.galenos.2019.81594 31650791 PMC6823586

[B59] Heckenlively J. R. Aptsiauri N. Nusinowitz S. Peng C. Hargrave P. A. (1996). Investigations of antiretinal antibodies in pigmentary retinopathy and other retinal degenerations. Trans. Am. Ophthalmol. Soc. 94, 179–200. discussion 200-6. 8981696 PMC1312095

[B60] Heckenlively J. R. Ferreyra H. A. (2008). Autoimmune retinopathy: A review and summary. Semin. Immunopathol. 30 (2), 127–134. 10.1007/s00281-008-0114-7 18408929

[B61] Heckenlively J. R. Jordan B. L. Aptsiauri N. (1999). Association of antiretinal antibodies and cystoid macular edema in patients with retinitis pigmentosa. Am. J. Ophthalmol. 127 (5), 565–573. 10.1016/s0002-9394(98)00446-2 10334350

[B62] Heckenlively J. R. Lundy S. K. (2018). “Autoimmune retinopathy: An immunologic cellular-driven disorder,” in Retinal degenerative diseases (Springer), 193–201. 10.1007/978-3-319-75402-4_24 29721944

[B63] Heckenlively J. R. Lundy S. K. (2018). Autoimmune retinopathy: An immunologic cellular-driven disorder. Adv. Exp. Med. Biol. 1074, 193–201. 10.1007/978-3-319-75402-4_24 29721944

[B64] Heckenlively J. R. Solish A. M. Chant S. M. Meyers-Elliott R. H. (1985). Autoimmunity in hereditary retinal degenerations. II. Clinical studies: Antiretinal antibodies and fluorescein angiogram findings. Br. J. Ophthalmol. 69 (10), 758–764. 10.1136/bjo.69.10.758 4052361 PMC1040734

[B65] Hogewind B. F. Zijlstra C. Klevering B. J. Hoyng C. B. (2008). Intravitreal triamcinolone for the treatment of refractory macular edema in idiopathic intermediate or posterior uveitis. Eur. J. Ophthalmol. 18 (3), 429–434. 10.1177/112067210801800318 18465727

[B66] Hollingsworth T. J. Gross A. K. (2020). Innate and autoimmunity in the pathogenesis of inherited retinal dystrophy. Cells 9 (3), 630. 10.3390/cells9030630 32151065 PMC7140441

[B67] Hollingsworth T. J. New D. D. Giorgianni F. Lenchik N. I. Beranova-Giorgianni S. Gerling I. C. (2015). Peptidylarginine deiminase (PAD4) expression and citrullination levels in normal and mouse retinas and in murine models of late (Sod1-/-) and early-onset (Tyro3-/-, Axl-/-, Mertk-/- or TAM mice) retinal degeneration. Invest. Ophthalmol. Vis. Sci. 56. E-Abstract 4636.

[B68] Hollingsworth T. J. Radic M. Z. Beranova-Giorgianni S. Giorgianni F. Wang Y. Iannaccone A. (2018). Murine retinal citrullination declines with age and is mainly dependent on peptidyl arginine deiminase 4 (PAD4). Invest. Ophthalmol. Vis. Sci. 59 (10), 3808–3815. 10.1167/iovs.18-24118 30073354 PMC6074612

[B69] Hollingsworth T. J. Radic M. Z. Giorgianni F. Beranova-Giorgianni S. Koirala D. Wang Y. (2017). Peptidylarginine deiminase 4 (PAD4) is the primary mediator of retinal citrullination in mice. Invest. Ophthalmol. Vis. Sci. 58. [E-Abstract no. assignement in progress]. 10.1167/iovs.18-24118 PMC6074612 30073354

[B70] Hong Y. Li H. Sun Y. Ji Y. (2020). A review of complicated cataract in retinitis pigmentosa: Pathogenesis and cataract surgery. J. Ophthalmol. 2020, 6699103. 10.1155/2020/6699103 33489339 PMC7803180

[B71] Iannaccone A. Alekseev O. Adamus G. (2021). Recognizing, characterizing, and managing inflammation in inherited retinal degenerations: Treatable, visually impactful complications beyond cystoid macular edema RD2021 Meeting. Nashville.

[B72] Iannaccone A. Giorgianni F. New D. D. Hollingsworth T. J. Umfress A. C. Alhatem A. H. (2015). Circulating autoantibodies in age-related macular degeneration recognize human macular tissue antigens implicated in autophagy, immunomodulation, and protection from oxidative stress and apoptosis. PLoS ONE 10 (12), e0145323. 10.1371/journal.pone.0145323 26717306 PMC4696815

[B73] Iannaccone A. Hollingsworth T. J. Koirala D. New D. D. Lenchik N. I. Beranova-Giorgianni S. (2017). Retinal pigment epithelium and microglia express the CD5 antigen-like protein, a novel autoantigen in age-related macular degeneration. Exp. Eye Res. 155, 64–74. 10.1016/j.exer.2016.12.006 27989757 PMC5796423

[B74] Iannaccone A. Man D. Waseem N. Jennings B. J. Ganapathiraju M. Gallaher K. (2006). Retinitis pigmentosa associated with rhodopsin mutations: Correlation between phenotypic variability and molecular effects. Vis. Res. 46 (27), 4556–4567. 10.1016/j.visres.2006.08.018 17014888

[B75] Iannaccone A. Neeli I. Krishnamurthy P. Lenchik N. I. Wan H. Gerling I. C. (2012). Autoimmune biomarkers in age-related macular degeneration: A possible role player in disease development and progression. Adv. Exp. Med. Biol. 723, 11–16. 10.1007/978-1-4614-0631-0_2 22183309

[B76] Iannaccone A. Radic M. Z. (2019). Increased protein citrullination as a trigger for resident immune system activation, intraretinal inflammation, and promotion of anti-retinal autoimmunity: Intersecting paths in retinal degenerations of potential therapeutic relevance. Adv. Exp. Med. Biol. 2019 (1185), 175–179. 10.1007/978-3-030-27378-1_29 31884608

[B77] Iannaccone A. Rispoli E. Vingolo E. M. Onori P. Steindl K. Rispoli D. (1995). Correlation between Goldmann perimetry and maximal electroretinogram response in retinitis pigmentosa. Doc. Ophthalmol. 90, 129–142. 10.1007/BF01203333 7497885

[B78] Iannaccone A. (2003). “Usher syndrome: Correlation between visual field size and maximal ERG response b-wave amplitude,” in Retinal degenerations: Mechanisms and experimental therapy. Adv exp med biol. 533. Editors LaVail M. M. Hollyfield J. G. Anderson R. E. (New York: Plenum Publishers), 123–131. 10.1007/978-1-4615-0067-4_16 15180256

[B79] Iannaccone A. Vingolo E. M. Tanzilli P. Del Beato P. Pannarale M. R. (Editors) (1994). Long-term results of a pilot study on thymopentin in the treatment of retinitis pigmentosa: Pathophysiological considerations [Italian] IV national congress of the Italian association for ocular pharmacology (AISFO) (Catania, Italy: Mediconsult). Isola di Capri.

[B80] Iannaccone A. Vingolo E. M. Tanzilli P. Del Beato P. Pannarale M. R. (1994). “Long-term results of a pilot study on thymopentin in the treatment of retintitis pigmentosa: Pathophysiological considerations [Italian],” in The IV national congress of the Italian association for ocular pharmacology (AISFO) (Catania: MediConsult), 461–473.

[B81] Jacobson S. G. Yagasaki K. Feuer W. J. Roman A. J. (1989). Interocular asymmetry of visual function in heterozygotes of X-linked retinitis pigmentosa. Exp. Eye Res. 48, 679–691. 10.1016/0014-4835(89)90009-2 2737262

[B82] Jain R. Ferrante P. Reddy G. T. Lightman S. (2005). Clinical features and visual outcome of intermediate uveitis in children. Clin. Exp. Ophthalmol. 33 (1), 22–25. 10.1111/j.1442-9071.2005.00938.x 15670074

[B83] Jones J. Francis P. (2009). Ophthalmic utility of topical bromfenac, a twice-daily nonsteroidal anti-inflammatory agent. Expert Opin. Pharmacother. 10 (14), 2379–2385. 10.1517/14656560903188425 19735215

[B84] Juthani V. V. Clearfield E. Chuck R. S. (2017). Non-steroidal anti-inflammatory drugs versus corticosteroids for controlling inflammation after uncomplicated cataract surgery. Cochrane Database Syst. Rev. 7 (7), CD010516. 10.1002/14651858.CD010516.pub2 28670710 PMC5580934

[B85] Kanie T. Jackson P. K. (2021). Connecting autoimmune disease to Bardet-Biedl syndrome and primary cilia. EMBO Rep. 22 (2), e52180. 10.15252/embr.202052180 33511755 PMC7857418

[B86] Karasu B. (2020). Short-term outcomes of subtenon triamcinolone acetonide injections in patients with retinitis pigmentosa-associated cystoid macular edema unresponsive to carbonic anhydrase inhibitors. Int. Ophthalmol. 40 (3), 677–687. 10.1007/s10792-019-01228-z 31773389

[B87] Khani S. C. Nielsen L. Vogt T. M. (1998). Biochemical evidence for pathogenicity of rhodopsin kinase mutations correlated with the oguchi form of congenital stationary night blindness. Proc. Natl. Acad. Sci. U. S. A. 95, 2824–2827. 10.1073/pnas.95.6.2824 9501174 PMC19653

[B88] Khurana R. N. Bansal A. S. Chang L. K. Palmer J. D. Wu C. Wieland M. R. (2017). Prospective evaluation of a sustained-release dexamethasone intravitreal implant for cystoid macular edema in quiescent uveitis. Retina 37 (9), 1692–1699. 10.1097/IAE.0000000000001406 27893624

[B89] Kim J. E. (2006). Intravitreal triamcinolone acetonide for treatment of cystoid macular edema associated with retinitis pigmentosa. Retina 26 (9), 1094–1096. 10.1097/01.iae.0000254897.86389.a5 17151506

[B90] Koop A. Ossewaarde A. Rothova A. (2013). Peripheral multifocal chorioretinitis: Complications, prognosis and relation with sarcoidosis. Acta Ophthalmol. 91 (6), 492–497. 10.1111/j.1755-3768.2012.02483.x 22863241

[B91] Ksantini M. Lafont E. Bocquet B. Meunier I. Hamel C. P. (2012). Homozygous mutation in MERTK causes severe autosomal recessive retinitis pigmentosa. Eur. J. Ophthalmol. 22 (4), 647–653. 10.5301/ejo.5000096 22180149

[B92] Kyger M. Worley A. Adamus G. (2013). Autoimmune responses against photoreceptor antigens during retinal degeneration and their role in macrophage recruitment into retinas of RCS rats. J. Neuroimmunol. 254 (1-2), 91–100. 10.1016/j.jneuroim.2012.10.007 23110938 PMC3534925

[B93] Lemos Reis R. F. Moreira-Goncalves N. Estrela Silva S. E. Brandao E. M. Falcao-Reis F. M. (2015). Comparison of topical dorzolamide and ketorolac treatment for cystoid macular edema in retinitis pigmentosa and Usher's syndrome. Ophthalmologica 233 (1), 43–50. 10.1159/000368052 25428176

[B94] Liu X. Feng B. Vats A. Tang H. Seibel W. Swaroop M. (2020). Pharmacological clearance of misfolded rhodopsin for the treatment of RHO-associated retinitis pigmentosa. FASEB J. 34 (8), 10146–10167. 10.1096/fj.202000282R 32536017 PMC7688577

[B95] Mansour A. M. Sheheitli H. Kucukerdonmez C. Sisk R. A. Moura R. Moschos M. M. (2018). Intravitreal dexamethasone implant in retinitis pigmentosa-related cystoid macular edema. Retina 38 (2), 416–423. 10.1097/IAE.0000000000001542 28221257

[B96] Marmor M. F. (1990). Hypothesis concerning carbonic anhydrase treatment of cystoid macular edema: Example with epiretinal membrane. Arch. Ophthalmol. 108 (11), 1524–1525. 10.1001/archopht.1990.01070130026013 2244830

[B97] McCulloch D. L. Marmor M. F. Brigell M. G. Hamilton R. Holder G. E. Tzekov R. (2015). ISCEV Standard for full-field clinical electroretinography (2015 update). Doc. Ophthalmol. 130 (1), 1–12. 10.1007/s10633-014-9473-7 25502644

[B98] McMurtrey J. J. Tso M. O. M. (2018). A review of the immunologic findings observed in retinitis pigmentosa. Surv. Ophthalmol. 63 (6), 769–781. 10.1016/j.survophthal.2018.03.002 29551596

[B99] Mercau M. E. Akalu Y. T. Mazzoni F. Gyimesi G. Alberto E. J. Kong Y. (2023). Inflammation of the retinal pigment epithelium drives early-onset photoreceptor degeneration in Mertk-associated retinitis pigmentosa. Sci. Adv. 9 (3), eade9459. 10.1126/sciadv.ade9459 36662852 PMC9858494

[B100] Miguel-Escuder L. Olate-Perez A. Sala-Puigdoners A. Moll-Udina A. Figueras-Roca M. Navarro-Angulo M. J. (2023). Intravitreal fluocinolone acetonide implant for the treatment of persistent post-surgical cystoid macular edema in vitrectomized eyes. Eur. J. Ophthalmol. 33 (1), NP23–NP27. 10.1177/11206721211046718 34836464

[B101] Mitchell J. Balem F. Tirupula K. Man D. Dhiman H. K. Yanamala N. (2019). Comparison of the molecular properties of retinitis pigmentosa P23H and N15S amino acid replacements in rhodopsin. PLoS ONE 14 (5), e0214639. 10.1371/journal.pone.0214639 31100078 PMC6524802

[B102] Miyake Y. Goto S. Ota I. Ichikawa H. (1984). Vitreous fluorophotometry in patients with cone-rod dystrophy. Br. J. Ophthalmol. 68 (7), 489–493. 10.1136/bjo.68.7.489 6733074 PMC1040388

[B103] Nakazawa M. Wada Y. Fuchs S. Gal A. Tamai M. (1997). Oguchi disease: Phenotypic characteristics of patients with the frequent 1147DELA mutation in the arrestin gene. Retina 17, 17–22. 10.1097/00006982-199701000-00004 9051837

[B104] Newsome D. A. Michels R. G. (1988). Detection of lymphocytes in the vitreous gel of patients with retinitis pigmentosa. Am. J. Ophthalmol. 105 (6), 596–602. 10.1016/0002-9394(88)90050-5 3377040

[B105] Nguyen X. T. Thiadens A. Fiocco M. Tan W. McKibbin M. Klaver C. C. W. (2023). Outcome of cataract surgery in patients with retinitis pigmentosa. Am. J. Ophthalmol. 246, 1–9. 10.1016/j.ajo.2022.10.001 36252678

[B106] Park S. Lim L. T. Gavin M. P. (2013). Topical steroidal and nonsteroidal antiinflammatory drugs for the treatment of cystoid macular edema in retinitis pigmentosa. Retin Cases Brief. Rep. 7 (2), 134–136. 10.1097/ICB.0b013e31825a300f 25390804

[B107] Park U. C. Park J. H. Ma D. J. Cho I. H. Oh B. L. Yu H. G. (2020). A randomized paired-eye trial of intravitreal dexamethasone implant for cystoid macular edema in retinitis pigmentosa. Retina 40 (7), 1359–1366. 10.1097/IAE.0000000000002589 31166248

[B108] Patil L. Lotery A. J. (2014). Coat's-like exudation in rhodopsin retinitis pigmentosa: Successful treatment with an intravitreal dexamethasone implant. Eye (Lond). 28 (4), 449–451. 10.1038/eye.2013.314 24458202 PMC3983638

[B109] Percopo C. M. Hooks J. J. Shinohara T. Caspi R. Detrick B. (1990). Cytokine-mediated activation of a neuronal retinal resident cell provokes antigen presentation. J. Immunol. 145 (12), 4101–4107. 10.4049/jimmunol.145.12.4101 2147935

[B110] Perry H. D. Donnenfeld E. D. (2006). An update on the use of ophthalmic ketorolac tromethamine 0.4%. Expert Opin. Pharmacother. 7 (1), 99–107. 10.1517/14656566.7.1.99 16370927

[B111] Petrushkin H. Rogers D. Pavesio C. (2018). The use of topical non-steroidal anti-inflammatory drugs for uveitic cystoid macular edema. Ocul. Immunol. Inflamm. 26 (5), 795–797. 10.1080/09273948.2016.1269931 28080174

[B112] Pichi F. Nucci P. Baynes K. Lowder C. Y. Srivastava S. K. (2017). Sustained-release dexamethasone intravitreal implant in juvenile idiopathic arthritis-related uveitis. Int. Ophthalmol. 37 (1), 221–228. 10.1007/s10792-016-0265-9 27221263

[B113] Rispoli E. Vingolo E. M. Iannaccone A. (1991). Thymopentin in Retinitis Pigmentosa. Evaluation of its possible therapeutical effects after 18 months of treatment, preliminary results. New Trends Ophthalmol. 6, 235–241.

[B114] Rispoli E. Vingolo E. M. Iannaccone A. (1990). “Thymopentin (Timunox) in retinitis pigmentosa: Evaluation of its therapeutical effects after 18 months of treatment. Preliminary results,” in XXVI international congress of ophthalmology (ICO) (Singapore. March 18-24.

[B115] Rossetto J. D. Nascimento H. Fernandes D. D. Belfort R. Jr. Muccioli C. (2015). Treatment of cystoid macular edema secondary to chronic non-infectious intermediate uveitis with an intraocular dexamethasone implant. Arq. Bras. Oftalmol. 78 (3), 190–193. 10.5935/0004-2749.20150049 26222112

[B116] Saade J. S. Istambouli R. AbdulAal M. Antonios R. Hamam R. N. (2021). Bromfenac 0.09% for the treatment of macular edema secondary to noninfectious uveitis. Middle East Afr. J. Ophthalmol. 28 (2), 98–103. 10.4103/meajo.meajo_134_21 34759667 PMC8547661

[B117] Sandberg M. A. Weigel-DiFranco C. Rosner B. Berson E. L. (1996). The relationship between visual field size and electroretinogram amplitude in retinitis pigmentosa. Invest. Ophthalmol. Vis. Sci. 37, 1693–1698. 8675413

[B118] Saraiva V. S. Sallum J. M. Farah M. E. (2003). Treatment of cystoid macular edema related to retinitis pigmentosa with intravitreal triamcinolone acetonide. Ophthalmic Surg. Lasers Imaging 34 (5), 398–400. 10.3928/1542-8877-20030901-11 14509465

[B119] Schaal Y. Hondur A. M. Tezel T. H. (2016). Subtenon triamcinolone for cystoid macular edema due to retinitis pigmentosa unresponsive to oral acetazolamide. Can. J. Ophthalmol. 51 (4), e113–e115. 10.1016/j.jcjo.2015.12.021 27521675

[B120] Schallhorn J. M. Niemeyer K. M. Browne E. N. Chhetri P. Acharya N. R. (2018). Difluprednate for the treatment of uveitic cystoid macular edema. Am. J. Ophthalmol. 191, 14–22. 10.1016/j.ajo.2018.03.027 29580977

[B121] Schilling H. Heiligenhaus A. Laube T. Bornfeld N. Jurklies B. (2005). Long-term effect of acetazolamide treatment of patients with uveitic chronic cystoid macular edema is limited by persisting inflammation. Retina 25 (2), 182–188. 10.1097/00006982-200502000-00011 15689809

[B122] Scorolli L. Morara M. Meduri A. Reggiani L. B. Ferreri G. Scalinci S. Z. (2007). Treatment of cystoid macular edema in retinitis pigmentosa with intravitreal triamcinolone. Arch. Ophthalmol. 125 (6), 759–764. 10.1001/archopht.125.6.759 17562986

[B123] Sen E. S. Dick A. D. Ramanan A. V. (2015). Uveitis associated with juvenile idiopathic arthritis. Nat. Rev. Rheumatol. 11 (6), 338–348. 10.1038/nrrheum.2015.20 25825278

[B124] Slabaugh M. A. Herlihy E. Ongchin S. van Gelder R. N. (2012). Efficacy and potential complications of difluprednate use for pediatric uveitis. Am. J. Ophthalmol. 153 (5), 932–938. 10.1016/j.ajo.2011.10.008 22265149

[B125] Steinmetz R. L. Fitzke F. W. Bird A. C. (1991). Treatment of cystoid macular edema with acetazolamide in a patient with serpiginous choroidopathy. Retina 11 (4), 412–415. 10.1097/00006982-199110000-00008 1813958

[B126] Studsgaard A. Clemmensen K. O. Nielsen M. S. (2022). Intravitreal fluocinolone acetonide 0.19 mg (Iluvien®) for the treatment of uveitic macular edema: 2-year follow-up of 20 patients. Graefes Arch. Clin. Exp. Ophthalmol. 260 (5), 1633–1639. 10.1007/s00417-021-05504-6 34851465

[B127] Stump M. Guo D. F. Rahmouni K. (2023). T cell-specific deficiency in BBSome component BBS1 interferes with selective immune responses. Am. J. Physiol. Regul. Integr. Comp. Physiol. 324 (2), R161–R170. 10.1152/ajpregu.00243.2022 36534590 PMC9844976

[B128] Sudhalkar A. Kodjikian L. Borse N. (2017). Intravitreal dexamethasone implant for recalcitrant cystoid macular edema secondary to retinitis pigmentosa: A pilot study. Graefes Arch. Clin. Exp. Ophthalmol. 255 (7), 1369–1374. 10.1007/s00417-017-3660-7 28378252

[B129] Tanner V. Kanski J. J. Frith P. A. (1998). Posterior sub-Tenon's triamcinolone injections in the treatment of uveitis. Eye (Lond). 12 (4), 679–685. 10.1038/eye.1998.168 9850264

[B130] Tranos P. G. Wickremasinghe S. S. Stangos N. T. Topouzis F. Tsinopoulos I. Pavesio C. E. (2004). Macular edema. Surv. Ophthalmol. 49 (5), 470–490. 10.1016/j.survophthal.2004.06.002 15325193

[B131] Travassos A. Fishman G. Cunha-Vaz J. G. (1985). Vitreous fluorophotometry studies in retinitis pigmentosa. Graefes Arch. Clin. Exp. Ophthalmol. 222 (4-5), 237–240. 10.1007/BF02133687 3979850

[B132] Tsyklauri O. Niederlova V. Forsythe E. Prasai A. Drobek A. Kasparek P. (2021). Bardet-Biedl Syndrome ciliopathy is linked to altered hematopoiesis and dysregulated self-tolerance. EMBO Rep. 22 (2), e50785. 10.15252/embr.202050785 33426789 PMC7857422

[B133] Veritti D. Sarao V. De Nadai K. Chizzolini M. Parmeggiani F. Perissin L. (2020). Dexamethasone implant produces better outcomes than oral acetazolamide in patients with cystoid macular edema secondary to retinitis pigmentosa. J. ocular Pharmacol. Ther. official J. Assoc. Ocular Pharmacol. Ther. 36 (3), 190–197. 10.1089/jop.2018.0153 31886707

[B134] Vingolo E. M. Iannaccone A. Forte R. Tanzilli P. Sciò F. (Editors) (1993). “Thymopentin in the treatment of retinitis pigmentosa: Results after a three-year follow-up,” Retinitis pigmentosa present knowledge and outlook (Naples, Italy: Edizioni Liviana Medicina).

[B135] Vingolo E. M. Iannaccone A. Rispoli E. Pannarale L. Amodeo S. Pannarale M. R. (1993). “Three-year experience in the treatment of retinitis pigmentosa with thymopentin,” in 4th meeting of the schepens international society (Hong Kong. March 30-April 2.

[B136] Viswanathan S. Frishman L. J. Robson J. G. Harwerth R. S. Smith E. L. III (1999). The photopic negative response of the macaque electroretinogram: Reduction by experimental glaucoma. Invest. Ophthalmol. Vis. Sci. 40, 1124–1136. 10235545

[B137] Wolfensberger T. J. Aptsiauri N. Godley B. Downes S. Bird A. C. (2000). Antiretinale Antikörper assoziiert mit zystoidem Makulaödem1. Klin. Monatsblatter fur Augenheilkd. 216 (5), 283–285. 10.1055/s-2000-10561 10863693

[B138] Wolfensberger T. J. Mahieu I. Jarvis-Evans J. Boulton M. Carter N. D. Nogradi A. (1994). Membrane-bound carbonic anhydrase in human retinal pigment epithelium. Invest. Ophthalmol. Vis. Sci. 35 (9), 3401–3407. 8056514

[B139] Wolfensberger T. J. (1999). The role of carbonic anhydrase inhibitors in the management of macular edema. Doc. Ophthalmol. 97 (3-4), 387–397. 10.1023/a:1002143802926 10896355

[B140] Wong C. W. Metselaar J. M. Storm G. Wong T. T. (2021). A review of the clinical applications of drug delivery systems for the treatment of ocular anterior segment inflammation. Br. J. Ophthalmol. 105 (12), 1617–1622. 10.1136/bjophthalmol-2020-315911 33127826

[B141] Wu L. Arevalo J. F. Hernandez-Bogantes E. Roca J. A. (2012). Intravitreal infliximab for refractory pseudophakic cystoid macular edema: Results of the pan-American collaborative retina study group. Int. Ophthalmol. 32 (3), 235–243. 10.1007/s10792-012-9559-8 22484726

[B142] Xiong W-H. Duvoisin R. M. Adamus G. Jeffrey B. G. Gellman C. Morgans C. W. (2013). Serum TRPM1 autoantibodies from melanoma associated retinopathy patients enter retinal on-bipolar cells and attenuate the electroretinogram in mice. PLoS One 8 (8), e69506. 10.1371/journal.pone.0069506 23936334 PMC3731326

[B143] Yamamoto S. Sippel K. C. Berson E. L. Dryja T. P. (1997). Defects in the rhodopsin kinase gene in the Oguchi form of stationary night blindness. Nat. Genet. 15 (2), 175–178. 10.1038/ng0297-175 9020843

[B144] Yang P. Lockard R. Titus H. Hiblar J. Weller K. Wafai D. (2020). Suppression of cGMP-dependent photoreceptor cytotoxicity with mycophenolate is neuroprotective in murine models of retinitis pigmentosa. Invest. Ophthalmol. Vis. Sci. 61 (10), 25. 10.1167/iovs.61.10.25 PMC7441375 32785677

[B145] Yoshii M. Murakami A. Akeo K. Nakamura A. Shimoyama M. Ikeda Y. (1998). Visual function and gene analysisin a family with oguchi's disease. Ophthalmic Res. 30, 394–401. 10.1159/000055501 9731122

