# Supplementary material for: The double-edged sword of inflammation in inherited retinal degenerations: Clinical and preclinical evidence for mechanistically and prognostically impactful but treatable complications
Source: Front Cell Dev Biol. 2023 Apr 13;11:1177711. doi: 10.3389/fcell.2023.1177711 (PMC10135873; doi:10.3389/fcell.2023.1177711)
Supplement: Supplementary file 1 [file Table1.DOCX]

**Visual function and clinical/imaging criteria used to identify subjects with IRD-like
presentations associated with inflammatory complications**

| ***Clinical/imaging criteria associated with  suspected inflammatory complications*** | ***Visual function criteria associated with  suspected inflammatory complications*** |
| --- | --- |
| - Absence of waxy pallor on DFE - Presence of outright overt disc hyperemia and/or swelling on DFE - Thickened RNFL on OCT imaging (macular and/or PP scans) - Late leakage and/or staining on FA of the disc, arcades, macula, peripheral vascular, focal or disseminated - Presence of significant CME (especially if unresponsive / only partially responsive to CAIs) - Disseminated and/or flame-shaped retinal exudates on DFE (as frequently seen in primary and paraneoplastic AIR and AIR/ARRON patients) - Disseminated chorioretinal peripheral nummular “punched-out” atrophic lesions on DFE | - Late-onset visual function loss or sudden acceleration in the vision loss process - BCVA less than potential predicted by foveal EZ preservation not explained by media opacities or other factors (e.g., amblyopia) - Worse VF loss than predicted by the level of ffERG reduction - Asymmetry in VF loss not explained by other   factors   - Enlarged blind spots or centro-cecal scotomas on VF testing not associated with PP atrophy or other PP chorioretinal lesions - Delayed pattern VEPs despite normal or reasonable (20/25-20/30) BCVA - Electronegative ffERGs with evidence of post-receptoral dysfunction and/or (when measurable) disproportionate RGC-driven response (PhNR) reduction |

DFE, dilated fundus examination; RNFL, retinal nerve fiber layer; OCT, optical coherence tomography; PP, peripapillary; FA, fluorescein angiography; CAIs, carbonic anhydrase inhibitors; AIR, autoimmune retinopathy; ARRON, autoimmune-related retinopathy and optic neuropathy; BCVA, best corrected visual acuity; EZ, ellipsoid zone; VF, visual field; VEPs, visual evoked potentials; ffERGs full-field electroretinogram; RGC, retinal ganglion cells
