# Supplementary material for: The double-edged sword of inflammation in inherited retinal degenerations: Clinical and preclinical evidence for mechanistically and prognostically impactful but treatable complications
Source: Front Cell Dev Biol. 2023 Apr 13;11:1177711. doi: 10.3389/fcell.2023.1177711 (PMC10135873; doi:10.3389/fcell.2023.1177711)
Supplement: Supplementary file 2 [file Table2.DOCX]

**Supplemental Table.**

| **Gene** | **Total AAb-positive per gene** | **Total genotyped** | **Positive % all IRD-associated AINR** | **Positive % per number of genotype-confirmed patients** |
| --- | --- | --- | --- | --- |
| ***ABCA4*** | 8 | 125 | 6.3% | 6% |
| ***BBS1*** | 5 | 16 | 3.9% | 31% |
| ***BEST1*** | 1 | 9 | 0.8% | 11% |
| ***C2orf71 (PCARE)*** | 2 | 2 | 1.6% | 100% |
| ***CDHR1*** | 2 | 2 | 1.6% | 100% |
| ***CEP78*** | 1 | 1 | 0.8% | 100% |
| ***CERKL*** | 1 | 1 | 0.8% | 100% |
| ***CLN3*** | 1 | 9 | 0.8% | 11% |
| ***CRB1*** | 7 | 36 | 5.5% | 19% |
| ***CRX*** | 2 | 3 | 1.6% | 67% |
| ***EYS*** | 13 | 22 | 10.2% | 59% |
| ***HMCN1*** | 1 | 1 | 0.8% | 100% |
| ***IFT172*** | 1 | 1 | 0.8% | 100% |
| ***IMPDH1*** | 1 | 1 | 0.8% | 100% |
| ***KLHL7*** | 3 | 3 | 2.4% | 100% |
| ***MERTK**** | 2 | 2 | 1.6% | 100% |
| ***MYO7A*** | 1 | 13 | 0.8% | 8% |
| ***NR2E3*** | 5 | 10 | 3.9% | 50% |
| ***PDE6B*** | 2 | 12 | 1.6% | 17% |
| ***PRCD*** | 1 | 1 | 0.8% | 100% |
| ***PRPF31*** | 7 | 10 | 5.5% | 70% |
| ***PRPF8*** | 3 | 14 | 2.4% | 21% |
| ***PRPH2*** | 8 | 22 | 6.3% | 36% |
| ***RDH12*** | 1 | 7 | 0.8% | 14% |
| ***RHO*** | 11 | 33 | 8.7% | 33% |
| ***RP1*** | 3 | 12 | 2.4% | 25% |
| ***RPGR*** | 3 | 71 | 2.4% | 4% |
| ***TUB*** | 2 | 3 | 1.6% | 67% |
| ***USH1C*** | 2 | 4 | 1.6% | 50% |
| ***USH2A*** | 23 | 69 | 18.1% | 35% |
| **TOTAL** | **127** | **515** | – | ­– |

* Count does not include the 3/3 previously reported AAb-positive *MERTK* patients (see main text)
